# The Effect of Computerized Alerts on Prescribing and Patient Outcomes: A Systematic Review

**DOI:** 10.1055/a-2620-3244

**Published:** 2025-10-17

**Authors:** Brian G. Bell, Adam Khimji, Basharat Hussain, Anthony J. Avery

**Affiliations:** 1 NIHR Greater Manchester Patient Safety Research Collaboration, University of Nottingham, Nottingham, United Kingdom; 2 Birmingham Community Healthcare NHS Foundation Trust, Birmingham, United Kingdom; 3 University of Nottingham, Nottingham, United Kingdom

**Keywords:** computerized alerts, prescribing, patient outcomes, human–computer interaction, alerting-drug–drug interaction

## Abstract

**Objective:**

In recent years, there has been an expansion in the literature on the effects of computerized alerts on prescribing and patient outcomes. The aim of our study was to examine the impact of these systems on clinician prescribing and patient outcomes.

**Methods:**

We searched three databases (Medline, Embase, and PsychINFO) for studies that had been conducted since 2009 and included studies that examined the effects of alerts at the point of prescribing. We extracted data from 69 studies.

**Results:**

Most studies reported a beneficial effect on prescribing of computerized alerts ( *n* = 58, 84.1%), including all studies ( *n* = 4) that used passive alerts. Seven of the 10 studies that reported on patient outcomes showed a beneficial effect. Both randomized controlled trials (RCTs) and non-RCTS showed beneficial effects on prescribing across a range of different types of alerts. In 43 studies, it was possible to ascertain the effects of different types of alerts; the interventions that were most frequently associated with improvements in prescribing were drug-laboratory alerts (9/11; 81.8%); dose range checking (6/7; 85.7%); formulary alerts (8/9; 88.9%), and drug-allergy alerts (4/4; 100%). However, most of the studies did not satisfy the quality criteria.

**Conclusion:**

Most of the studies found a beneficial effect of computerized alerts on prescribing. We have also shown that these benefits are apparent for a range of different types of alerts. These findings support the continued development, implementation, and evaluation of computerized alerts for prescribing.

## Background and Significance

Although the prescribing of medication aims to provide appropriate and effective treatment for patients, medication errors are also responsible for morbidity and mortality. Errors can produce harm, with 2.4 to 3.6% of hospital admissions caused by adverse drug events (ADEs), 69% of which would have been preventable. 1 Silva et al 2 conducted a systematic review in which they reported that the worldwide hospitalization rate from ADEs ranged between 9.7 and 383.0 per 100,000 population and the mortality rate ranged between 0.1 and 7.88 per 100,000 population. Ayalew et al 3 found that antithrombotic drugs, antihypertensive drugs, analgesics, anti-diabetics, antipsychotics, and anti-neoplastic drugs were mainly responsible for drug-related hospitalizations with a third of these hospitalizations definitely preventable, and more than 40% probably preventable.

Recent systematic reviews have looked at the effects of computerized physician order entry (CPOE) and clinical decision support systems (CDSS) on reducing the occurrence of ADEs. Pallares et al 4 reported that the use of CPOE and clinical decision support (CDS) reduced prescribing errors in hospitals and Gohari et al 5 found that the use of these systems significantly decreased the rate of errors and reduced ADEs in emergency departments. However, there is evidence for their effect on patient outcomes. A systematic review found that patient care improved 6 from the use of these systems, although Cerqueira et al 7 concluded that the effects of prescribing alerts on patient outcomes were unclear.

Medication errors that lead to harm can potentially be reduced by computerized CDS that uses appropriate alerts and prompts. A previous systematic review from 2009 showed the benefits of CDS software alerts at the point of prescribing in reducing hazardous prescriptions. 8 Most of the types of CDS software identified (23/27) improved prescribing and/or reduced error rates. Of the four alert types that examined clinical outcomes, three had a positive and statistically significant impact. In two studies, the use of CDS-software alerts was found to be cost-saving. However, more recent systematic reviews have not found benefits or have produced mixed results. Bayoumi et al 9 reported that drug-laboratory alerts did not reduce ADEs or improve clinical outcomes. A systematic review published in 2017 10 found that computerized alerts improved prescribing, although this study was less conclusive than the earlier study 8 with only a little more than half of the studies (53%) showing a beneficial effect on prescribing; only two studies reported on patient outcomes and no improvement was found in these.

Several recent studies 11 12 13 14 15 have found a beneficial effect of computerized alerts on prescribing while other studies have not. 16 17 18 19 Bakker et al 11 reported that drug–drug interaction alerts reduced the prescribing of high-risk drug combinations; Blaga et al 12 found that opioid dosing for ophthalmologic conditions was reduced by alerts; Nelson et al 13 concluded that their alerting system led to an increase in co-prescribing naloxone; Rabbani et al 14 found that alerts reduced free-text prescribing, and Srikumar et al 15 reported an increase in naloxone prescriptions after alert implementation. However, Desmedt et al 16 found that alerts did not significantly reduce inappropriate drug dosages for patients with renal failure; Hansen et al 17 concluded that alerts did not reduce antibiotic prescribing; Rolfzen et al 18 reported that alerts did not reduce opioid prescribing; and Smith et al 19 stated that alerts had no effect on the co-prescribing of opioids and benzodiazepines. Therefore, we decided to update an earlier systematic review 8 to address a gap in the literature to provide up-to-date knowledge of the impact of different types of alerts on clinician's prescribing and patient outcomes.

## Materials and Methods

**Fig. 1:**
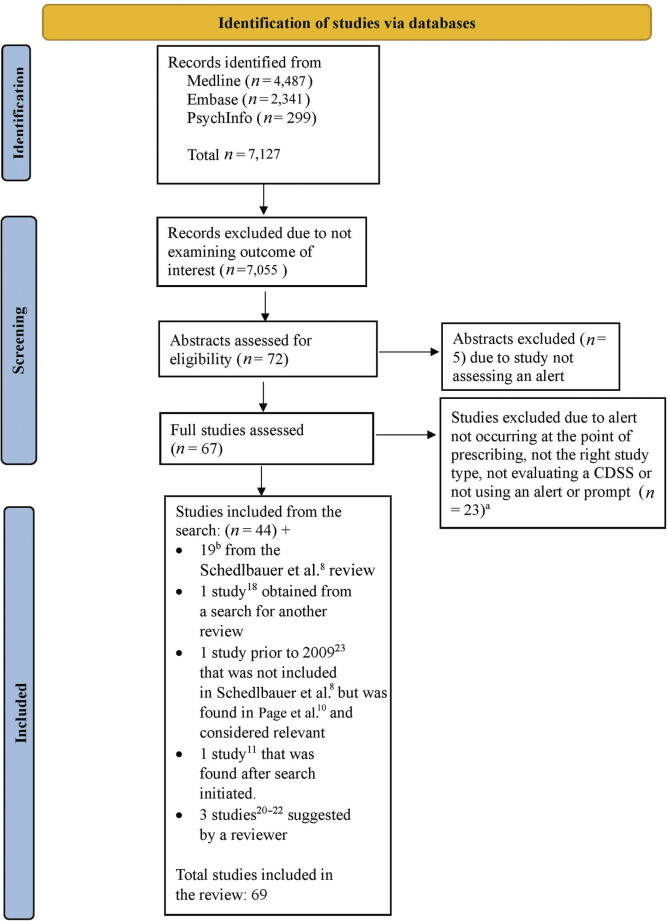
Flow chart showing the sequence of steps in identifying relevant papers. ^a^ Excluded due to alert not occurring at the point of prescribing ( *n* = 17, 73.9%), not the right study type (4, 17.4%), not evaluating a clinical decision support system ( *n* = 1, 4.3%), or not using an alert or prompt ( *n* = 1, 4.3%). ^b^ One study that was included in Schedlbauer et al 8 was excluded because it was not an alert at the point of prescribing.

We searched three databases (Medline, Embase, and PsychINFO) for studies that had been conducted since 2009. We included only studies reported in English. The search terms are provided in Supplementary Appendix 1 (available in the online version only). These searches were conducted for studies from January 2009 to September 2023 for Embase, Medline, and PsychInfo. A previous literature review 10 was also searched and any relevant references were added. We also included one study 18 that was not found in the searches and was obtained from the search for another systematic review. One other study was subsequently added that a colleague found, which was published after our search dates, 11 and three studies were added based on a reviewer's recommendations. 20 21 22 The flow chart outlining the sequence of steps in identifying and selecting papers is shown in Fig. 1 .

Studies were included if they met four criteria:

1. The study was either a randomized controlled trial (RCT), a before/after study, or a time series analysis.
2. The study had to examine the effect of a reminder, alert, or prompt on the behavior of clinicians (doctors, nurses, or other healthcare professionals) involved in the prescribing of medication.
3. The intervention had to consist of an alert or reminder at the point of prescribing.
4. The study was included if it examined changes in prescribing related to clinically relevant outcomes, such as a reduction in medication errors or risk/harm to patients.

All settings were considered including both primary and secondary care. The searches were uploaded into Rayyan software. 23 The titles and abstracts were independently examined by two reviewers who then met to resolve disagreements. When agreement could not be reached, the senior investigator, with considerable expertise in prescribing safety, made the final decision.

We extracted the data from the 69 studies, which can be seen in Supplementary Appendix 2 (available in the online version only), and this included 19 of the 20 studies from the 2009 review. 8 As seen in Supplementary Appendix 2 (available in the online version only), we used the classification scheme from this systematic review, 8 which included setting (primary or secondary care), country, study design, duration, participants, the alert objectives, alert design, outcome, and whether the alert had a beneficial effect on prescribing and patient outcomes. We added whether the alert was interruptive or passive, the alert category, which was taken from the review by Page et al, 10 and whether the outcome could be attributed to a particular category of alert. Interruptive alerts are those alerts that require a response from the clinician, which interrupts workflow, whereas passive alerts are those that appear but do not require a response from the clinician.

Quality assessment of the studies was done using the approach of the earlier review. 8 We assessed whether RCTs satisfied the following criteria: allocation concealment, blinding of the generator (the person who allocated the people to the control and intervention groups), whether the providers were blind to the allocation, whether losses to follow-up were reported, and whether there was a power calculation. For the before/after studies and time series analysis, the criteria were: whether the seasonal influence was accounted for (by having an adequate period of time before and after the intervention, typically a year), whether the assessment of outcomes was similar in the intervention and control periods, and whether the groups were similar in the intervention and control periods.

We ran descriptive statistics for the studies but did not perform a meta-analysis because the studies were heterogeneous. The software package that we used was SPSS version 27. 24

## Results

Table 1 shows the characteristics of the 69 studies contained in this review. Almost two-thirds ( *n* = 45) of the studies, 13 14 15 17 18 19 20 21 22 25 26 27 28 29 30 31 32 33 34 35 36 37 38 39 40 41 42 43 44 45 46 47 48 49 50 51 52 53 54 55 56 57 58 59 were conducted in the United States with a little over 10% ( *n* = 7) being done in the Netherlands. 11 60 61 62 63 64 65 A little more than half of the studies ( *n* = 37) were before/after studies, 12 13 15 16 17 20 22 25 26 28 29 31 32 34 38 40 42 43 46 54 57 58 59 60 61 62 63 65 66 67 68 69 70 71 72 73 74 about a quarter was a time series analysis ( *n* = 19) 19 21 30 33 37 39 44 45 47 48 50 52 53 56 75 76 77 78 79 and just under 20% were an RCT ( *n* = 13). 11 14 18 27 35 36 41 49 51 55 64 80 81 Over three-quarters of the studies ( *n* = 53) were performed in secondary care 11 12 13 14 15 16 18 20 21 22 25 26 28 29 30 31 32 33 34 37 38 39 40 41 42 43 44 45 46 47 48 50 51 55 56 57 60 61 62 63 65 66 67 69 70 71 72 73 75 76 77 with a little under 20% ( *n* = 13) 19 35 36 49 52 53 54 59 64 68 79 80 81 being in primary care. Most of the studies reported interruptive alerts ( *n* = 56, 81.2%) 11 12 13 14 15 16 17 19 20 21 22 25 26 27 28 31 32 33 35 36 37 38 39 40 41 42 43 44 46 47 49 50 51 52 53 54 55 56 57 60 61 63 64 65 67 68 69 70 71 74 75 76 78 79 80 81

**Table 1:** Study characteristics

Characteristic	*n* (%)
Country	
Australia	1 (1.5) 66
Belgium	1 (1.5) 16
Canada	1 (1.5) 80
France	2 (2.9) 75 76
Greece	1 (1.5) 67
Ireland	1 (1.5) 68
Italy	1 (1.5) 81
Japan	2 (2.9) 69 77
The Netherlands	7 (10.1) 11 60 61 62 63 64 65
South Korea	1 (1.5) 70
Spain	3 (4.45) 71 72 73
Switzerland	1 (1.5) 78
Taiwan	1 (1.5) 79
Thailand	1 (1.5) 74
United States	45 (65.2) 13 14 15 17 18 19 20 21 22 25 26 27 28 29 30 31 32 33 34 35 36 37 38 39 40 41 42 43 44 45 46 47 48 49 50 51 52 53 54 55 56 57 58 59
Study design	
Time series analysis	19 (27.5) 19 21 30 33 37 39 44 45 47 48 50 52 53 56 75 76 77 78 79
Before/after	37 (53.6) 12 13 15 16 17 20 22 25 26 28 29 31 32 34 38 40 42 43 46 54 57 58 59 60 61 62 63 65 66 67 68 69 70 71 72 73 74
RCT	13 (18.8) 11 14 18 27 35 36 41 49 51 55 64 80 81
Location	
Primary care	13 (18.8) 19 35 36 49 52 53 54 59 64 68 79 80 81
Secondary care	53 (76.8) 11 12 13 14 15 16 18 20 21 22 25 26 28 29 30 31 32 33 34 37 38 39 40 41 42 43 44 45 46 47 48 50 51 55 56 57 60 61 62 63 65 66 67 69 70 71 72 73 75 76 77
Primary and secondary care	3 (4.3) 17 27 58
Type of Alert	
Interruptive	56 (81.2) 9 11 12 13 14 15 16 17 19 20 21 22 25 26 27 28 31 32 33 35 36 37 38 39 40 41 42 43 44 46 47 49 50 51 52 53 54 55 56 57 60 61 63 64 65 67 68 69 70 71 74 75 76 78 79 80 81
Passive	4 (5.8) 48 58 62 77
Interruptive and passive	2 (2.9) 45 59
Undetermined	7 (10.1) 18 29 30 34 66 72 73
Alert category a	
Drug laboratory alert	25 (36.2) 15 27 29 30 35 36 37 38 41 45 47 48 54 59 60 61 62 65 68 69 71 76 77 79 81
Dose range checking	25 (36.2) 12 13 15 16 21 25 28 32 33 34 36 41 42 45 47 50 62 63 66 69 70 71 75 76 79
Drug–drug interaction	25 (36.2) 11 13 15 19 21 22 28 29 30 34 35 40 41 44 50 55 56 59 61 63 71 72 73 79 81
Drug condition interaction	21 (30.4) 13 15 17 21 22 26 40 44 46 49 57 59 63 64 68 69 71 74 75 80 81
Formulary alert	17 (24.6) 14 15 22 26 31 40 42 43 44 47 52 53 56 58 70 78 80
Dose adjustment	14 (20.3) 16 18 25 27 33 36 42 47 50 62 63 67 70 79
Corollary order alert	10 (14.5) 13 21 22 34 41 56 60 61 63 70
Drug allergy interaction	7 (10.1) 25 28 29 30 34 50 63
Duplicate order	5 (7.2) 20 25 34 56 79
Intravenous to oral conversion	1 (1.4) 39
Alert category not clear	1 (1.4) 51

a Many studies used more than one type of alert.

The most common alert types were drug laboratory alerts ( *n* = 25, 36.2%%), 15 27 29 30 35 36 37 38 41 45 47 48 54 59 60 61 62 65 68 69 71 76 77 79 81 dose range checking ( *n* = 25, 36.2%), 12 13 15 16 21 25 28 32 33 34 36 41 42 45 47 50 62 63 66 69 70 71 75 76 79 and drug–drug interaction ( *n* = 25, 36.2%). 11 13 15 19 21 22 28 29 30 34 35 40 41 44 50 55 56 59 61 63 71 72 73 79 81 Table 2 shows the assessment of the quality of the studies that are included in this review. As can be seen in Table 2 , the studies did not, for the most part, satisfy the quality criteria. Only 15.4% ( *n* = 2) 11 27 of the RCTs used allocation concealment. For 61.5% ( *n* = 8), 11 14 27 35 51 55 80 81 the generator was blind to the allocation to control and treatment groups. In around a quarter of the studies (23.1%, *n* = 3) 18 41 64 the providers were blind to the allocation. Losses to follow-up were reported in 53.8% of studies ( *n* = 7) 11 41 49 55 64 80 81 and only a little more than half (53.8% *n* = 7) 11 18 27 35 64 80 81 provided a power calculation. For the before/after studies, 21.4% ( *n* = 12) 12 13 25 26 39 44 52 53 59 77 78 79 accounted for seasonal influences and a little more than half (51.8%, *n* = 29) 16 17 20 21 22 26 28 29 32 34 37 38 40 45 47 48 50 57 58 60 61 63 67 72 73 74 75 76 77 had similar groups in the intervention and control periods, although all ( *n* = 56, 100%) 12 13 15 16 17 19 20 21 22 25 26 28 29 30 31 32 33 34 37 38 39 40 42 43 44 45 46 47 48 50 52 53 54 56 57 58 59 60 61 62 63 65 66 67 68 69 70 71 72 73 74 75 76 77 78 79 assessed similar outcomes in the control and intervention periods.

**Table 2:** Quality assessment of studies

**Type of study**	***n*** **(%)**
RCTs	
Allocation concealment	
Yes	2 (15.4) 11 27
No	4 (30.8) 36 41 51 64
Unclear	7 (53.8) 14 18 35 49 55 80 81
Blinding of generator	
Yes	8 (61.5) 11 14 27 35 51 55 80 81
No	3 (23.1) 36 41 64
Unclear	2 (15.4) 18 49
Providers blind to allocation	
Yes	3 (23.1) 18 41 64
No	7 (53.8) 11 27 36 51 55 80 81
Unclear	3 (23.1) 14 35 49
Losses to follow-up reported	
Yes	7 (53.8) 11 41 49 55 64 80 81
No	2 (15.4) 35 51
Unclear	4 (30.8) 14 18 27 36
Power calculation	
Yes	7 (53.8) 11 18 27 35 64 80 81
No	6 (46.2) 14 36 41 49 51 55
Unclear	0 (0.0)
Before/after and time series analysis	
Seasonal influence accounted for	
Yes	12 (21.4) 12 13 25 26 39 44 52 53 59 77 78 79
No	40 (71.4) 15 16 17 19 28 29 30 31 32 33 34 37 38 40 42 43 45 46 47 48 50 54 56 57 58 60 61 62 63 65 66 68 69 70 71 72 73 74 75 76
Unclear	4 (7.1) 20 21 22 67
Assessment of outcomes similar	
Yes	56 (100.0) 12 13 15 16 17 19 20 21 22 25 26 28 29 30 31 32 33 34 37 38 39 40 42 43 44 45 46 47 48 50 52 53 54 56 57 58 59 60 61 62 63 65 66 67 68 69 70 71 72 73 74 75 76 77 78 79
No	0 (0.0)
Similar groups in control and intervention	
Yes	29 (51.8) 16 17 20 21 22 26 28 29 32 34 37 38 40 45 47 48 50 57 58 60 61 63 67 72 73 74 75 76 77
No	9 (16.1) 13 19 31 33 59 62 65 66 79
Unclear	18 (32.1) 12 15 25 30 39 42 43 44 46 52 53 54 56 68 69 70 71 78

Abbreviation: RCT, randomized controlled trial.

In Table 3 , we show the effect of computerized alerts on prescribing and patient outcomes by study type. Most of the RCTs ( *n* = 8, 61.6%) 11 14 27 36 51 55 80 81 and non-RCTs ( *n* = 50, 89.3%) 12 13 15 20 21 22 25 26 28 29 30 31 32 33 34 37 38 39 40 42 43 44 45 46 47 48 50 53 54 56 57 58 60 61 62 63 65 66 67 68 69 70 71 72 73 74 75 77 78 79 reported a beneficial effect on prescribing. Of the 11 studies that reported on patient outcomes ( Table 3 ), seven showed a beneficial effect ( *n* = 7, 10.1%); two RCTs 11 80 and five non-RCTs. 6 33 47 48 57 Two studies 54 66 did not find a statistically significant effect, one demonstrated 55 unintended consequences that were not associated with a detrimental outcome and one showed detrimental effects, 67 which consisted of a rise in surgical site infections, although the authors state that they remained within acceptable limits. Most (6/7; 85.7%) of the studies that found beneficial effects were done in secondary care and most (5/8; 62.5%) used interruptive alerts. The beneficial effects included a reduction in the length of stay in the intensive care unit, 11 a reduction in the risk of injury, 80 a reduction in serious and life-threatening ADEs, 30 a reduction in hospital stay, 33 fewer falls, 47 reduced renal impairment, 48 and a reduction in QTc interval prolongation, 57 which is a risk factor for cardiac arrest. In the study 55 that found unintended consequences, a nearly “hard stop” prescribing alert intended to reduce concomitant orders for warfarin and trimethoprim-sulfamethoxazole caused ”either a delay of treatment with trimethoprim-sulfamethoxazole when determined to be necessary for treatment or inadvertent warfarin discontinuation.”

**Table 3:** Effect on prescribing and patient outcomes by study type

Beneficial effect on prescribing reported	*n* (%)
RCTs	
No (not significant)	5 (38.5%) 18 35 41 49 64
Yes	8 (61.6%) 11 14 27 36 51 55 80 81
Before/after and time series analysis	
No (not significant)	5 (8.9%) 16 17 19 52 76
Yes a	50 (89.3%) 12 13 15 20 21 22 25 26 28 29 30 31 32 33 34 37 38 39 40 42 43 44 45 46 47 48 50 53 54 56 57 58 60 61 62 63 65 66 67 68 69 70 71 72 73 74 75 77 78 79
Yes and no b	1 (1.8%) 59
Beneficial effect on patient outcomes reported	
RCTs	
No c	1 (7.7%) 55
Yes	2 (15.4%) 11 80
Not reported	10 (76.9%) 14 18 27 35 36 41 49 51 64 81
Before/after and time series analysis	
No d	1 (1.8%) 67
No (not significant)	2 (3.6%) 54 66
Yes e	5 (8.9%) 6 33 47 48 57
Not reported	48 (85.7%) 12 13 15 16 17 19 20 21 22 25 26 28 29 31 32 34 37 38 39 40 42 43 44 45 46 50 52 53 56 58 59 60 61 62 63 65 68 69 70 71 72 73 74 75 76 77 78 79

Abbreviation: RCT, randomized controlled trial.

a Three studies that reported a beneficial effect did not report a *p* -value.

b One study found both positive and negative effects.

c One RCT ended early due to unintended consequences that were not detrimental.

d One study showed a detrimental effect on patient outcomes.

e One of the studies that reported a beneficial effect did not report a *p* -value.

When the effect on prescribing was examined by whether the study used an interruptive or passive alert, we found that of the 12 RCTs that used interruptive alerts, 8 (66.7%) 11 14 27 36 51 55 80 81 found a beneficial effect on prescribing and four (33.3%) 35 41 49 64 found no effect. None of the RCTs used passive alerts. For the before/after and time series studies, of those studies that used interruptive alerts, 38 (88.4%) 12 13 15 21 22 25 26 28 31 32 33 37 38 39 40 42 43 44 46 47 50 53 54 56 57 60 61 63 65 67 68 69 70 71 74 75 78 79 found a beneficial effect on prescribing and five (11.6%) 16 17 19 52 76 found no effect. All four of the before/after and time series studies that used passive alerts found a beneficial effect onprescribing. 48 58 62 77

We examined the 43 (62.3%) studies where either only one type of alert was evaluated or it was possible to ascertain the effects of different types of alert on prescribing; the results are shown in Table 4 (these studies are identified in the last column of Appendix 2 ). We report only on changes in prescribing as few of these studies reported on patient outcomes. Nine (20.9%) of these studies were RCTs 11 14 18 27 36 41 49 55 64 and 34 (79.1%) were before/after or time series studies. 12 17 19 20 25 29 30 31 32 37 38 39 42 43 46 48 50 52 53 54 56 57 58 59 60 61 65 66 67 72 73 74 77 78 Across the study types, the interventions that were most frequently associated with improvements in prescribing were drug-laboratory alerts (9/11; 81.8%) 27 36 37 38 48 54 60 65 77 including two of the three RCTs; 27 36 dose range checking (6/7; 85.7%); 12 25 32 50 56 66 formulary alerts (8/9; 88.9%) 14 31 42 43 53 56 58 78 and drug-allergy alerts (4/4; 100%). 25 29 30 50 Of the other intervention types reported in at least four studies, improvements were noted for dose-adjustment (5/6; 83.3%), 25 27 50 56 67 corollary orders (3/4; 75%), 56 60 61 drug–drug interaction alerts (5/10; 50%) 11 55 59 72 73 and drug-condition alerts (4/8; 50%). 46 57 59 74

**Table 4:** The effect of alert category on prescribing by study type

**Alert category**	**Beneficial effect on prescribing**	**No effect on prescribing**
RCTs	Number of alerts	Number of alerts
Drug laboratory alert	2 27 36	1 41
Dose range checking	0	1 41
Drug–drug interaction	2 11 55	1 41
Drug condition interaction	0	2 49 64
Formulary alert	1 14	0
Dose adjustment	1 27	1 18
Corollary order alert	0	1 41
Drug allergy interaction	0	0
Duplicate order	0	0
Intravenous to oral conversion	0	0
Before/after and time series analysis		
Drug laboratory alert	7 37 38 48 54 60 65 77	1 59
Dose range checking	6 12 25 32 50 56 66	0
Drug–drug interaction	3 59 72 73	4 19 29 30 50
Drug condition interaction a	4 46 57 59 74	2 17 59
Formulary alert	7 31 42 43 53 56 58 78	1 52
Dose adjustment	4 25 50 56 67	0
Corollary order alert	3 56 60 61	0
Drug allergy interaction	4 25 29 30 50	0
Duplicate order	3 20 25 56	0
Intravenous to oral conversion	1 39	0

Abbreviation: RCT, randomized controlled trial.

a One of the studies 55 reported both a positive effect and no effect and was counted twice in this row.

b One study did not report a *p* -value.

## Discussion and Conclusion

We reviewed 69 studies on the effects of computer alerts and prompts on medication prescribing. Most of the alerts were interruptive and fell into one of four categories: drug laboratory alerts, dose range checking, drug–drug interaction, and drug–condition interaction. Almost all the studies reported a beneficial effect on prescribing and most of the studies that reported on patient outcomes reported a beneficial effect, although it should be noted that for one study, 55 a nearly “hard stop” prescribing alert intended to reduce concomitant orders for warfarin and trimethoprim-sulfamethoxazole caused clinically important treatment delays for four patients who needed immediate treatment. Another study 67 reported a rise in surgical site infections after an alert was implemented aimed at reducing the duration of antimicrobial chemoprophylaxis in cardiac surgery. We also note that the one study that examined renal dosing 48 provides compelling evidence in terms of patient outcomes and this may be because clinicians recognize the particular importance of dose adjustment in patients with renal impairment.

A strength of this study is the large number of studies we found ( *n* = 50), which provides a significant increase on the number of studies that Schedlbauer et al 8 ( *n* = 20) found with more than two-thirds of the studies published since 2009. Another strength was that we were able to categorize the alerts by whether they were interruptive or passive and the type of alert, such as drug condition interaction; we were able to separate the alerts into various categories and examine the effects of alert category on prescribing outcome.

Nevertheless, most of the studies did not satisfy all the quality criteria that we used. This is not surprising given the challenges of employing rigorous approaches such as “allocation concealment” and “providers being blind to allocation” in studies where clinicians are likely to know (or to notice) if a new CDSS has been introduced or if changes have been made to an existing system. Also, most (84%) of the studies did not report the effects of alerts on patient outcomes, and we cannot assume that any improvements in prescribing described in these studies would translate to improved outcomes. In addition, there were relatively few studies from outside the United States and from primary care, so we have less evidence that the benefits described in our review apply to these settings. In many studies, we could not attribute the prescribing outcome to a particular category of alert. For example, even though 25 studies used drug–drug interaction alerts, for only 10 studies could the outcome be unequivocally ascribed to this alert. Finally, there may have been publication bias with studies with positive findings being more likely to be published than those with negative findings.

Our results are similar to the previous review from 2009, 8 in that most of the studies found a beneficial effect on prescribing, but our review is a significant advance because it contains more than three times the number of studies (69 vs. 20). Our findings differ from the 2017 review by Page et al 10 where only a little more than half of the studies reported a beneficial effect on prescribing. Page et al 10 suggested this was due to more recent studies showing less favorable results, but our review did not bear this out; the more recent studies identified from our current review indicate a balance in favor of the computerized alerts.

The most important factor in whether prescribing alerts affect prescribing is probably related to the design of the alerts and their incorporation into the workflow. In this regard, Marcilly et al 82 developed a list of usability principles to improve the design of computer alerting systems for prescribing. The principles include reducing over-alerting by considering the clinical context, encouraging professional collaboration among clinicians, presenting alerts in a timely and seamless fashion that improves workflow, providing alerts that contain relevant data, helping the user to understand the system's limitations, and providing tools that allow the user to take action based on the alert. These principles have been translated into a tool called TEMAS (tool for evaluating medication alerting systems) that assesses hospital alerting systems. 83 Unfortunately, although most of the participants said the items in the TEMAS were easy to understand, it was not easy to use with low to moderate inter-rater reliability (0.26–0.46) Most of the participants (88%) in this study had negative or neutral opinions of the alerts in their hospital. In a related vein, Marcilly et al 84 have identified challenges that hospital pharmacists encounter when using CDSSs for medication reviews. They identified four challenges, which include not being able to identify the most important alerts, slow response of the CDSS, design of the CDSS not reflecting how a medication review is conducted, and alerts being viewed as invalid or not relevant. A recent study 85 of antibiotic prescribing in the emergency department found that users of a new CDSS strongly preferred the new system to the old electronic health records (EHR), which did not have decision support. In developing the new CDSS, the authors conducted usability testing, which suggests that incorporating this element into the design of the system may improve satisfaction among users.

While we have been able to describe the studies in our review in some detail (see Supplementary Appendix 2 [available in the online version only]) the methods described in the studies often do not provide sufficient information for an analysis of the impact of particular design features. Nevertheless, it makes sense to take into account recognized usability principles in the future design of CDS. In particular, it is important to pay close attention to potential unintended consequences of CDS 55 where a near “hard stop” alert resulted in clinically important delays to treatment for patients. Also, while all the studies involving passive alerts reported in our review showed positive results, none of these studies were randomized trials which limits any conclusions that can be made about these alerts. Design principles suggest that interruptive alerts are likely to be more effective than passive alerts and these are the only types of alert supported with evidence from randomized trials. Nevertheless, alert fatigue, in which the clinician overrides numerous alerts that are considered irrelevant, needs to be taken into account. In this regard, a systematic review 86 found that CPOE alerts had low positive predictive value, which means that many of the alerts were not considered clinically relevant. van der Sijs et al 87 reported that between 49 and 96% of drug safety alerts were overridden by clinicians.

Another point of note is that our review coincides with the passage of the American Recovery and Reinvestment Act of 2009, which prompted the adoption of commercial EHRs in the United States and the replacement of locally developed EHRs. This may have led to deleterious effects due to the transition from one system to another. We examined the two studies that spanned this period and found that one study 49 reported that the effect of the CDSS did not affect prescribing while the other 57 found that prescribing improved, so we found no evidence of a deleterious effect in the studies that we reviewed.

While the results of this review are positive overall, several suggestions arise for future research. First, wherever possible it is important for researchers to conduct randomized trials that fit standard quality criteria or high-quality quasi-experimental studies. Second, there is a need for more studies that report patient outcomes. Third, there is an important need for more studies from primary care and from outside the United States. In this regard, the full functionality of CDSS systems in primary care may need to be improved. Co et al 88 found in a sample of outpatient clinics from the United States that many CDSS capabilities were not implemented. Fourthly, it would be helpful for researchers to provide more detail on design principles used in alert creation so that future systematic reviews can elucidate which of these are most important for effectiveness.

In conclusion, the clear majority of studies, including randomized trials, showed positive effects on prescribing from drug-laboratory alerts, dose range checking, formulary alerts, dose adjustment, drug-allergy alerts, and corollary order alerts, this covers much of the important functionality of alerting systems, although it should be noted that in many cases we could not attribute the prescribing outcome to a particular category of alert. In addition, where patient outcomes were examined, the majority of these also reported positive effects. This suggests that improvements in prescribing are translated into benefits for patients and should encourage providers to continue to invest in these systems.

## Clinical Relevance Statement

Computerized alerts improve prescribing, but their effect on patient outcomes, such as hospitalizations, is unclear. Although only 11 studies reported patient outcomes, most found a beneficial effect. This is clearly an area for future research along with an examination of the unintended consequences of decision support.

## Multiple-Choice Questions

1. The beneficial effects of alerts on patient outcomes include:
  - Reduction in emergency department visits
  - Fewer falls
  - Reduction in mortality
  - Reduction in blood pressure
  **Correct Answer:** The correct answer is option b. The correct answer is fewer falls as the other options were not among those listed above. However, few studies looked at patient outcomes, so this is clearly an area for further research as future studies may show effects on all of these outcomes. The research may be of limited value if it doesn't look at the broader issue of patient health.
2. Alerts that were most frequently associated with improvements in prescribing were:
  - Drug laboratory alerts
  - Duplicate order alerts
  - Intravenous to oral conversion alerts
  - Drug condition alerts
  **Correct Answer:** The correct answer is option a. The correct answer is drug laboratory alerts where nine studies found a beneficial effect on prescribing. However, fewer studies used the other alerts so further research is needed to determine whether these types of alerts have a beneficial effect on prescribing.
