# Supplementary material for: The Effect of Computerized Alerts on Prescribing and Patient Outcomes: A Systematic Review
Source: Appl Clin Inform. 2025 Oct 17;16(5):1381–92. doi: 10.1055/a-2620-3244 (PMC12534129; doi:10.1055/a-2620-3244)
Supplement: Supplementary file 1 — Supplementary Material [file 10-1055-a-2620-3244_27253336.pdf]

Supplementary Appendix 1: Search terms

Medline	
1.	"Drug Utilization Review"/
2.	(e adj2 prescri$).mp. [mp = title, book title, abstract, original title, name of substance word, subject heading word, floating sub-heading word, keyword heading word, organism supplementary concept word, protocol supplementary concept word, rare disease supplementary concept word, unique identifier, synonyms, population supplementary concept word, anatomy supplementary concept word]
3.	(elec$ adj prescri$).mp. [mp = title, book title, abstract, original title, name of substance word, subject heading word, floating sub-heading word, keyword heading word, organism supplementary concept word, protocol supplementary concept word, rare disease supplementary concept word, unique identifier, synonyms, population supplementary concept word, anatomy supplementary concept word]
4.	elec$ prescri$.mp. [mp = title, book title, abstract, original title, name of substance word, subject heading word, floating sub-heading word, keyword heading word, organism supplementary concept word, protocol supplementary concept word, rare disease supplementary concept word, unique identifier, synonyms, population supplementary concept word, anatomy supplementary concept word]
5.	electronic prescribing.mp.
6.	exp Drug Therapy, Computer-Assisted/
7.	exp Prescriptions/ or exp Drug Prescriptions/ or prescriptions.mp.
8.	or/1-7
9.	(design$ adj2 alert$).mp. [mp = title, book title, abstract, original title, name of substance word, subject heading word, floating sub-heading word, keyword heading word, organism supplementary concept word, protocol supplementary concept word, rare disease supplementary concept word, unique identifier, synonyms, population supplementary concept word, anatomy supplementary concept word]
10.	alert$ design$.mp.
11.	computer$ prompt$.mp.
12.	decision support.mp.
13.	drug alert$.mp. [mp = title, book title, abstract, original title, name of substance word, subject heading word, floating sub-heading word, keyword heading word, organism supplementary concept word, protocol supplementary concept word, rare disease supplementary concept word, unique identifier, synonyms, population supplementary concept word, anatomy supplementary concept word]
14.	exp Adverse Drug Reaction Reporting Systems/
15.	exp Decision Making/
16.	exp Decision Support Systems, Clinical/
17.	exp Decision Support Techniques/
18.	exp Drug Therapy, Computer-Assisted/
19.	exp Information Systems/
20.	exp Reminder Systems/
21.	prompt$ system$.mp.
22.	system prompt$.mp.
23.	or/9-22
24.	8 and 23
25.	limit 24 to (English language and humans and yr = "2009–Current")
**Embase**	
1.	"Drug Utilization Review"/
2.	(e adj2 prescri$).mp. [mp = title, abstract, heading word, drug trade name, original title, device manufacturer, drug manufacturer, device trade name, keyword heading word, floating subheading word, candidate term word]
3.	(elec$ adj prescri$).mp. [mp = title, abstract, heading word, drug trade name, original title, device manufacturer, drug manufacturer, device trade name, keyword heading word, floating subheading word, candidate term word]
4.	elec$ prescri$.mp. [mp = title, abstract, heading word, drug trade name, original title, device manufacturer, drug manufacturer, device trade name, keyword heading word, floating subheading word, candidate term word]

(*Continued*)

 © 2025. The Author(s).

Supplementary Appendix 1: (*Continued*)

5.	electronic prescribing.mp.
6.	exp Drug Therapy, Computer-Assisted/
7.	exp Prescriptions/ or exp Drug Prescriptions/ or prescriptions.mp.
8.	or/1-7
9.	(design$ adj2 alert$).mp. [mp = title, abstract, heading word, drug trade name, original title, device manufacturer, drug manufacturer, device trade name, keyword heading word, floating subheading word, candidate term word]
10.	alert$ design$.mp.
11.	computer$ prompt$.mp.
12.	decision support.mp.
13.	drug alert$.mp. [mp = title, abstract, heading word, drug trade name, original title, device manufacturer, drug manufacturer, device trade name, keyword heading word, floating subheading word, candidate term word]
14.	exp Adverse Drug Reaction Reporting Systems/
15.	exp Decision Making/
16.	exp Decision Support Systems, Clinical/
17.	exp Decision Support Techniques/
18.	exp Drug Therapy, Computer-Assisted/
19.	exp Information Systems/
20.	exp Reminder Systems/
21.	prompt$ system$.mp.
22.	system prompt$.mp.
23.	or/9-22
24.	8 and 23
25.	limit 24 to (human and English language and "remove Medline records" and yr = "2009–Current")
PsychInfo	
1.	"Drug Utilization Review"/
2.	(e adj2 prescri$).mp. [mp = title, abstract, heading word, table of contents, key concepts, original title, tests & measures, mesh word]
3.	(elec$ adj prescri$).mp. [mp = title, abstract, heading word, table of contents, key concepts, original title, tests & measures, mesh word]
4.	elec$ prescri$.mp. [mp = title, abstract, heading word, table of contents, key concepts, original title, tests & measures, mesh word]
5.	electronic prescribing.mp.
6.	exp Drug Therapy, Computer-Assisted/
7.	Prescriptions/ or Drug Prescriptions/ or Prescriptions.mp.
8.	or/1-7
9.	(design$ adj2 alert$).mp. [mp = title, abstract, heading word, table of contents, key concepts, original title, tests & measures, mesh word]
10.	alert$ design$.mp.
11.	computer$ prompt$.mp.
12.	decision support.mp.
13.	drug alert$.mp. [mp = title, abstract, heading word, table of contents, key concepts, original title, tests & measures, mesh word]
14.	exp Adverse Drug Reaction Reporting Systems/
15.	exp Decision Making/
16.	exp Decision Support Systems, Clinical/
17.	exp Decision Support Techniques/

 © 2025. The Author(s).

Supplementary Appendix 1: (*Continued*)

18.	exp Drug Therapy, Computer-Assisted/
19.	exp Information Systems/
20.	exp Reminder Systems/
21.	prompt$ system$.mp.
22.	system prompt$.mp.
23.	or/9-22
24.	8 and 23
25.	limit 24 to yr = "2009–Current"
26.	limit 25 to (human and English language)

 © 2025. The Author(s).

Supplementary Appendix 2: Summary table of the studies included in the review

Author<br>Year<br>Setting<br>Country	Study design/methods	Duration and participants	Type of alert/alert category	Alert objectives	Alert, reminder, or prompt and alert design	Outcomes/results	Beneficial effect on prescribing?	Beneficial effect on patient outcomes?	Can the outcome be attributed to a particular category of alert?
Agostini<br>2007<br>Secondary Care<br>United States	Before/After	12 mo<br>2002/2003<br>patients ≥65	Interruptive/Drug–Condition Interaction, Formulary alert	To reduce the prescribing of sedative-hypnotic drugs (diphenhydramine, diazepam, lorazepam, and trazodone). Increase the use of nonpharmacological methods for insomnia management	Reminder/Computer order-entry system, the Clinical Care Support System (Eclipsys Corporation). Each time diphenhydramine or diazepam was selected, the physician was exposed to up to 3 sequential screens. The first screen ascertained whether the drug was being prescribed as a sedative-hypnotic drug, if "yes" the clinician was directed to an educational reminder that reviewed potential adverse effects, pt's age, and recommendations for nonpharmacological and sedative-hypnotic medication use. If "no" the physician could order a recommended formulary medication or cancel the order. Picture given	≥1 sedative-hypnotics ordered for % of patients: 18% (B) to 15% (A); $p < 0.001$; OR = 0.82; (95% CI: 0.76–0.87); 18% reduction in sedative-hypnotic orders	Yes. 18% reduction in prescribing of sedative-hypnotics in the elderly	NR	No
Awdishu[a]<br>2016<br>Primary and Secondary Care<br>United States	Cluster RCT	10 mo<br>May 2012-March 2013<br>1,579 alerts for 254 physicians in intervention group<br>2,489 alerts for 260 physicians in the control group	Interruptive/Dose adjustment; Drug laboratory alert	Reduction of inappropriate medication orders for patients with acute or chronic kidney disease	Alert/For prospective medication alerts, if a medication was contraindicated for patients with a particular creatine clearance (CLcr) level, the alert fired as soon as the medication was selected. Look-back alerts were triggered for active medications when a threshold CLcr level was met and the provider accessed the order entry activity for that patient. For dose-adjusted medications, the alert fired after the order was signed, if the threshold CLcr level was met and the dose prescribed was in excess of the appropriate total daily dose	Prescribing orders were appropriately adjusted 17% of the time vs. 5.7% of the time in the intervention and control arms, respectively; (OR: 1.89, 95% CI, 1.45–2.47, $p < 0.0001$)	Yes. The intervention had a significant impact on the primary outcome (OR: 1.89, 95% CI, 1.45–2.47, $p < 0.0001$), a 20% increase in the rate of contraindicated medications discontinued or drug dosage adjustments for patients with kidney disease	NR	Yes
Bakker[d]<br>2024<br>Secondary Care<br>The Netherlands	RCT	12 mo<br>September 1, 2018, to September 1, 2019<br>5,534 patients in intervention group<br>4,353 patients in control group	Interruptive/Drug–drug interaction	Alerts for potential high-risk drug–drug interactions	Alert/A computerized algorithm, which defined a potential DDI as the administration of two drugs known to interact, administered within a 24-h interval. Only drugs that were actually administered were considered. Alerts for drug combinations assessed as high risk on the basis of a Delphi study were turned or left on, and alerts for drug combinations deemed as low yield were turned or left off. If ICUs used potential DDI alerts for drug combinations that were not evaluated in the Delphi study alerts for duplicate orders, or both, then these were left on because withholding these alerts might potentially negatively affect patient safety	The mean number of administered high-risk drug combinations per 1,000 drug administrations per patient was 26·2 (SD: 53·4) in the intervention group ($n = 5{,}534$), compared with 35·6 (65·0) in the control group ($n = 4{,}353$). Tailoring potential DDI alerts to the ICU led to a 12% decrease (95% CI: 5–18%; $p = 0.0008$) in the number of administered high-risk drug combinations per 1,000 drug administrations per patient, after adjusting for clustering and prognostic factors	Yes	Yes, there was a significant reduction in the length of stay in the intensive care unit for the intervention patient	Yes

 © 2025. The Author(s).

Supplementary Appendix 2: (*Continued*)

Author<br>Year<br>Setting<br>Country	Study design/methods	Duration and participants	Type of alert/alert category	Alert objectives	Alert, reminder, or prompt and alert design	Outcomes/results	Beneficial effect on prescribing?	Beneficial effect on patient outcomes?	Can the outcome be attributed to a particular category of alert?
Balasuriya[a]<br>2017<br>Secondary Care<br>United States	Before/After	4 mo<br>Before:<br>8,601 medication orders<br>July to August 2010<br>After:<br>12,482 medication orders<br>July to August 2011	Interruptive/Dose range checking, Drug allergy interaction, Drug–drug interaction	To reduce alert fatigue and to identify high-risk and frequently prescribed medications using both hard and soft alerts	Alert/Medication limits were set for each single dose prescribed. If a practitioner ordered a medication greater than its soft limit, the computer issued an alert describing the degree to which the prescribed dose exceeded normal parameters. This soft stop included usual medication dosing ranges and served as a "timeout" to reassess the dose of medication being ordered. A soft stop required acknowledgement with a comment, before allowing a provider to proceed with the medication order. The rationale given was submitted to the pharmacy with the medication order. In addition, when a soft or hard alert occurred, an e-mail was generated and automatically sent to clinical pharmacists for further monitoring. For additional protection, this system automatically generated a pharmacy consult when a hard alert order was submitted. If a practitioner prescribed a medication at a dose exceeding a hard limit, the order could not be submitted without discussion between an attending physician and a pharmacist. To reduce alert fatigue, if a provider ordered a medication that triggered a soft alert, that same provider could reorder the same medication dosage for that patient for the next 72 h without generating another alert. Safe buffer ranges were created for medications such as antibiotics so that doses could be exceeded within reasonable limits to account for rounding variances. These ranges were determined on the basis of the therapeutic index of the medication, with higher-risk medications having much smaller buffer ranges	Providers reduced doses to more appropriate levels after implementation ($p = 0.07$) and deleted orders more frequently ($p = 0.07$) after implementation. Fewer medication orders were submitted unmodified ($p = 0.007$)	Yes	NR	No

(*Continued*)

Supplementary Appendix 2: *(Continued)*

Author<br>Year<br>Setting<br>Country	Study design/methods	Duration and participants	Type of alert/alert category	Alert objectives	Alert, reminder, or prompt and alert design	Outcomes/results	Beneficial effect on prescribing?	Beneficial effect on patient outcomes?	Can the outcome be attributed to a particular category of alert?
Bates<br>1998<br>Secondary Care<br>United States	Before/After	2 time periods of 15 mo:<br>6 mo (B)<br>1993<br>9 mo (A)<br>1994/1995<br>In-patient<br>B 2,491<br>A 4,220	Undetermined/Drug–drug interaction; Drug–allergy interaction; Drug laboratory alert	To reduce serious medication errors, preventable ADEs, and potential ADEs. Definitions: Serious medication error = preventable ADEs + potential ADEs; All reported errors and ADEs are nonintercepted	Unclear/Introduction of a Physician Order Entry (POE) expected to reduce errors providing: menu of medication from formulary entry of dosage, route, and frequency by prescriber display of drug–drug interaction display of drug–allergy info display of relevant lab results (i.e., potassium with furosemide) follow-on order suggestions (i.e., aminoglycoside levels)	Events per 1,000 patient days: Serious medication errors fell by 55%. 10.7 (B) to 4.86 (A); $p = 0.01$; Potential ADEs fell by 84%: 5.99 (B) to 0.98 (A) $p = 0.002$; Preventable ADEs fell nonsignificantly by 17%. 4.69 (B) to 3.88 (A); $p = 0.37$; Nonpreventable ADE unchanged: 11.3 (B) to 11.3 (A); $p = 0.99$; Dose error decreased by 23%: 1.96 (B) to 1.51 (A); $p = 0.02$; Known allergy errors decreased by 56%: 0.65 (B) to 0.29 (A); $p = 0.009$; Drug–drug interactions error fell nonsignificantly by 40%: 0.41 (B) to 0.24 (A) $p = 0.89$	Yes. Serious Med. Error decreased by 55% nonintercepted potential ADE rate fell by 84% Dose error decreased by 23% Known allergy errors decreased by 56%. Drug–drug interactions error fell by 40%. Nonsignificant Preventable ADE rate fell by 17%. No Nonpreventable ADEs unchanged	NR	Yes
Bates<br>1999<br>Secondary Care<br>United States	Time series analysis	4 time periods<br>Baseline: 51 d October–November 1992<br>Period 1: 68 d October–December 1993<br>Period 2: 49 d November–December 1995<br>Period 3: 52 d, March–April 1997<br>Medication orders<br>Baseline: 10,070<br>Period 1: 15,025<br>Period 2: 13,139<br>Period 3: 14,352	Undetermined/Drug–drug interaction; Drug–allergy interaction; Drug laboratory alert	To reduce medication error rates	Unclear/Baseline: no automated decision support; orders written on paper; Period 1: complete order via POE, selection from standard list, basic drug–allergy and drug–drug interaction checking, some drug-lab notification; Period 2: Improved drug allergy checking; Period 3: Improved potassium ordering alerts, improved drug–drug interaction checking	Medication error rate per 1,000 pt d: baseline: 142; period 1: 51.2; period 2: 74.0; period 3: 26.6; $p < 0.0001$; Reduction by 81%; Serious medication error rate (combination of preventable ADEs and potential ADEs); Baseline: 7.6; Period 1: 7.3; Period 2: 1.7; Period 3: 1.1; $p = 0.0003$; Reduction by 86%; Preventable ADE:; 2.9 (base)-5.7 (1)-1.1 (2)-1.1 (3); $p = 0.05$; significant improvement by error type: dose, frequency, route, allergy; nonsignificant change by error type: drug–drug interaction	Yes. Substantial fall in medication errors (by 81%) and serious medication errors (86%) after the introduction of the complex alert system. This includes dose errors, frequency errors, route errors, and allergy-drug warnings. Nonsignificant Drug–drug interaction warnings did not improve significantly	Unclear, reduction in serious and life-threatening ADEs but no *p*-value reported (►**Table 4**, page 318)	Yes

 © 2025. The Author(s).

Supplementary Appendix 2: (*Continued*)

Author<br>Year<br>Setting<br>Country	Study design/methods	Duration and participants	Type of alert/alert category	Alert objectives	Alert, reminder, or prompt and alert design	Outcomes/results	Beneficial effect on prescribing?	Beneficial effect on patient outcomes?	Can the outcome be attributed to a particular category of alert?
Baypinar[a]<br>2017<br>Secondary Care<br>The Netherlands	Before/After	48 d:<br>August 25, 2015 to October 11, 2015<br>1,031 orders for methotrexate,<br>1,070 orders for bisphosphonates, and 13,412 orders for sodium-lowering drugs	Interruptive/Drug laboratory alert, Corollary order alert	The algorithms recommended co-prescribe folic or folinic acid with methotrexate, co-prescribe vitamin D with bisphosphonate, and alerting if sodium-lowering drugs were prescribed in patients with hyponatremia	Alert/The first algorithm shows a pop-up alert if oral or subcutaneous methotrexate was prescribed without co-prescription of folic or folinic acid. The second algorithm showed a pop-up alert if a bisphosphonate was prescribed, without co-prescription of vitamin D or an analog (colecalciferol, alfacalcidol, calcitriol, or dihydrotachysterol). The third algorithm showed a pop-up alert if one sodium-lowering drug is prescribed while the patient has a sodium level of 130 mmol/L or less, or if two or more sodium-lowering drugs are prescribed, while the patient has a sodium level of 135 mmol/L or less. Simultaneously with the introduction of the algorithm for hyponatremia, all drug–drug interaction alerts for combinations of drugs that lower sodium levels were filtered and not shown to the practitioners. If the physician decides to neglect the alert, a reason should be given with the buttons in the alert	Co-prescription of folic or folinic acid increased from 54 to 91% ($p = 0.014$), co-prescription of vitamin D or analogs increased from 11 to 40% ($p = 0.001$) and the number of stopped orders for sodium-lowering drugs increased from 3 to 14% ($p = 0.002$)	Yes	NR	Yes

(*Continued*)

Supplementary Appendix 2: (*Continued*)

Author<br>Year<br>Setting<br>Country	Study design/methods	Duration and participants	Type of alert/alert category	Alert objectives	Alert, reminder, or prompt and alert design	Outcomes/results	Beneficial effect on prescribing?	Beneficial effect on patient outcomes?	Can the outcome be attributed to a particular category of alert?
Becker[a]<br>2021<br>Secondary Care<br>The Netherlands	Before/After	3 mo for gastrointestinal prophylaxis alert, 1 y for intravenous drugs without plasma drug concentration, and 1.5 mo for the remaining 3 alerts<br>Before:<br>May 1, 2014 to August 1, 2014<br>May 1, 2014 to May 1, 2015<br>July 13, 2015 to August 24, 2015<br>After:<br>December 1, 2014 to March 1, 2015<br>June 1, 2015 to June 1, 2016<br>August 25, 2015 to October 11, 2015<br>All inpatients and outpatients at a teaching hospital in the Netherlands	Interruptive/Drug laboratory alert, Drug–drug interaction, Corollary order alert	1) Medication is ordered with an increased risk of gastrointestinal bleeding and, gastrointestinal prophylaxis is indicated based on age and medication use, but not prescribed.<br>2) Methotrexate is ordered for oral or subcutaneous administration, while no folic or folinic acid is prescribed.<br>3) bisphosphonate is ordered, while no colecalciferol, alfacalcidol, calcitriol, or dihydrotachysterol is prescribed.<br>4) One sodium-lowering drug is ordered while the sodium level is below 130 mmol/L or two sodium-lowering drugs are ordered concomitantly while the sodium level is below 135 mmol/L, with the advice to reconsider the sodium-lowering drug(s).<br>5) Intravenous vancomycin or gentamicin is ordered in a frequency other than once, and no recent plasma drug concentration was available nor a plasma drug concentration measurement was ordered	Alert/In the pop-up, the advice was given on how to manage the alert and if applicable the physician could order the recommended medication with two clicks. For the algorithm of measuring plasma concentrations for vancomycin or gentamicin, a drug concentration measurement could be ordered with two clicks. The algorithm for gastrointestinal prophylaxis in the advanced CDSS replaced all drug–drug interaction alerts from the basic CDSS for combinations of drugs with an increased risk of gastrointestinal bleeding. Similarly, the algorithm for hyponatremia in the advanced CDSS replaced all drug–drug interaction alerts from the basic CDSS for combinations of sodium-lowering drugs	For all five algorithms that were implemented, the compliance significantly increased and in three of the five algorithms, the compliance was above 80% after implementation. The acceptance rate of the advice given in the alert varied from 14 to 90%	Yes	NR	Yes

 © 2025. The Author(s).

Supplementary Appendix 2: (*Continued*)

Author<br>Year<br>Setting<br>Country	Study design/methods	Duration and participants	Type of alert/alert category	Alert objectives	Alert, reminder, or prompt and alert design	Outcomes/results	Beneficial effect on prescribing?	Beneficial effect on patient outcomes?	Can the outcome be attributed to a particular category of alert?
Blaga[a]<br>2022<br>Secondary Care<br>United States	Before/After	6 y<br>Before:<br>January 1, 2015 to December 19, 2017<br>After:<br>December 20, 2017 to November 3, 2021<br>8,014 patients	Interruptive/Dose range checking	To assess the efficacy of an opioid alert system in impacting opioid prescription practices in ophthalmology	Alert/Opioid prescriptions that exceeded a dosage of 30 morphine equivalent daily doses (MEDD) or a 7 d supply for adults prompted the system to fire an alert. The alert system applied to any discharge opioid medication that was written for the treatment of acute pain and was submitted through the institution's electronic medical record (EMR). Providers who at discharge attempted to prescribe an opioid for acute pain that exceeded 30 MEDD were met with a soft stop alert that could be accepted or dismissed. The alert did not prevent the provider from placing the order but required providers to document a reason for exceeding the restrictions for acute pain outpatient medications. This rule included prescriptions marked "PRN"(as needed). Providers who at discharge attempted to prescribe an opioid for acute pain without providing an associated diagnosis were not able to submit a prescription until a diagnosis was provided, known as a hard stop. Both the soft and hard stop alerts appeared as informational pop-up windows that described the Ohio opioid laws and their exceptions	The mean morphine equivalent daily dose (MEDD) per prescription decreased by 15.17 after implementation of the alert system ($p < 0.001$)	Yes	NR	Yes
Bogucki<br>2004<br>Secondary Care<br>United States	Before/After	4 mo<br>B: April 2003<br>May/June latent<br>A: July 2003<br>2,124 parenteral corticosteroid orders	Interruptive/Formulary alert	Reduce prescribing of methylprednisolone to overcome shortages of the medication	Reminder/Each time a prescription for methylprednisolone is entered, a message appears on the computer screen alerting the clinician to shortage and recommending 1 or 2 therapeutically equivalent drugs including dose conversions	Methylprednisolone orders: Reduction by 55%; 209 (B) to 112 (A); $p < 0.0001$; Dexamethasone orders: Increase by 12%; 702 (B) to 937 (A); $p < 0.0001$; Hydrocortisone orders: Increase by 49%; 59 (B) to 105 (A); $p < 0.0001$	Yes. Alert decreased orders for methylprednisolone by 55% and increased orders for therapeutically equivalent drugs	NR	Yes

(*Continued*)

 © 2025. The Author(s).

Supplementary Appendix 2: (*Continued*)

Author<br>Year<br>Setting<br>Country	Study design/methods	Duration and participants	Type of alert/alert category	Alert objectives	Alert, reminder, or prompt and alert design	Outcomes/results	Beneficial effect on prescribing?	Beneficial effect on patient outcomes?	Can the outcome be attributed to a particular category of alert?
Caro[a]<br>2014<br>Secondary Care<br>Spain	Before/After	3 mo in 2011<br>Before: 2,409 patients<br>After: 3,098 patients	Interruptive/Drug laboratory alert, Drug–drug interaction, Drug–condition interaction, Dose range checking	To quantify and analyze the impact of Electronic Alerts (EA) of the STOPP criteria included in an Assisted Electronic Prescription program (AEP) for minimizing Potentially Inappropriate Prescribing (PIP)	Alert/11 STOPP criteria were included in the assisted electronic prescription (AEP) program as eight electronic alerts. When the doctor prescribed a coded active ingredient according to one of the STOPP criteria, they were alerted, the prescriber could accept or reject the recommendation	After implementing the electronic alerts, an absolute risk reduction in the emergence of potentially inappropriate prescribing (PIP) of 0.17% (0.09–0.25%), a relative risk reduction of 39.23% (20.32–58.14%), and a relative risk of 0.61% (0.48–0.77%). The authors observed a PIP decline above 15% with all the EA, reaching up to 79% in the case of first-generation antihistamines	Yes	NR	No
Chen[a]<br>2022<br>Secondary Care<br>United States	Before/After	18 mo<br>Before:<br>January 1, 2018 to December 31, 2018<br>After:<br>June 28, 2020 to December 24, 2020<br>Before:<br>6,539 patients<br>After:<br>7,915 patients	Interruptive/Dose range checking	To automatically calculate the recommended daily maximum hydroxychloroquine dose based on ABW and flag excessively dosed prescriptions across a multicenter hospital system. In addition, the CDS's impact on the prevalence of excessive dosing based on the 2016 AAO recommendations	Alert/The tool identified the patient's most recent weight within the EMR and automatically calculated the maximum recommended daily dosage. In adults, the alert was triggered when any prescriber ordered hydroxychloroquine exceeding 5 mg/kg of ABW or 400 mg daily (the maximum dose for the treatment of systemic lupus erythematosus), whichever is lower. The alert user interface automatically displayed the maximum recommended daily dose calculated from a patient's weight, up to the maximum of 400 mg daily. The prescriber may choose to amend the prescription or to override the alert	After the intervention, the prevalence of excessive dosing decreased from 27.4 to 21.1% ($p < 0.001$) among all prescriptions and from 26.8 to 16.2% ($p < 0.001$) among new prescriptions. Daily doses exceeding 400 mg decreased from 0.8 to 0.02% ($p < 0.001$)	Yes	NR	Yes

Supplementary Appendix 2: (*Continued*)

Author<br>Year<br>Setting<br>Country	Study design/methods	Duration and participants	Type of alert/alert category	Alert objectives	Alert, reminder, or prompt and alert design	Outcomes/results	Beneficial effect on prescribing?	Beneficial effect on patient outcomes?	Can the outcome be attributed to a particular category of alert?
Chok[a]<br>2021<br>Secondary Care<br>Switzerland	Time series analysis	6 y<br>July 1, 2010 to June 30, 2016<br>4,100 hospital stays representing 5,549 prescriptions and 64,281 d of treatment	Interruptive/Formulary alert	The objectives of this study were to assess the effect of the electronic reminder on the number of days of therapy (DOTs) of meropenem, caspofungin, and voriconazole, and whether the number of prescriptions of these antimicrobials was affected by the intervention	Alert/The intervention consisted of the appearance of an alert in the patient's electronic chart in case of prescriptions of meropenem, voriconazole, or caspofungin. A pop-up window appeared for prescriptions and the following text was displayed: "Prescription of meropenem or new antifungal substances. You are prescribing voriconazole, caspofungin, or meropenem. Because of the risk of antibiotic resistance (meropenem) or their high costs (antifungals), the use of these substances at the University Hospital Zurich is restricted. Is there an indication according to the hospital antibiotic guidelines?" The physician then had to select "confirm prescription" or "cancel/stop prescription?" If the prescription was confirmed, a horizontal bar appeared in the warning/comment section of the electronic patient chart 72 h after the prescription, displaying the following text: "Comment on prescription of anti-infectives. The patient has been prescribed voriconazole, caspofungin, or meropenem for 72 h. According to the clinical course and the results, please assess whether (1) the current therapy should be continued, (2) the therapy may be streamlined (e.g., narrower spectrum) or (3) the therapy may be stopped. We advise you to involve the ID consultation service if you continue the current therapy (telephone contact provided)." The bar was displayed in orange color, indicating in the system that this information had to be validated by the treating physician, and turned green color if the physician confirmed having read the information	There was no significant change (−0.58, 95% CI: −1.48 to 0.33, $p = 0.2$) in the trend of prescriptions (slope of DOTs) when comparing the time before the alert, that is, July 2010 until June 2011, with the time after the alert, that is, July 2011 until June 2016. When analyzing prescriptions of the three drugs of interest separately, only DOTs/1,000 bed days for voriconazole revealed a significant decrease in slope ($p = 0.0017$) after the introduction of the alert. Only in the case of voriconazole, the intercept was significantly lower than 0 ($p < 0.001$) after the introduction of the alert and only the slope of voriconazole prescriptions per 1,000 bed days decreased significantly ($p < 0.001$) after the introduction of the alert	Yes	NR	Yes

(*Continued*)

 © 2025. The Author(s).

Supplementary Appendix 2: (*Continued*)

Author Year Setting Country	Study design/methods	Duration and participants	Type of alert/alert category	Alert objectives	Alert, reminder, or prompt and alert design	Outcomes/results	Beneficial effect on prescribing?	Beneficial effect on patient outcomes?	Can the outcome be attributed to a particular category of alert?
Chertow 2001 Secondary Care United States	Time series analysis	8 mo 4 × 2 mo 1997/1998 17,828 admissions 97,151 analyzable orders, 14,400 (15%) application-generated suggestions. 4,787 dosing alteration only 6,163 dosing and frequency alteration 253 warnings 27 substitute medication	Interruptive/Dose adjustment; Dose range checking	To increase the proportion of appropriate dose and frequency orders for patients with renal insufficiency	Reminder/Display of range of possible doses highlighting default dose. Adjustment of dose and frequency for patients with renal insufficiency based on incorporated lab results	Percentage of appropriate orders in patients with renal disorders: orders with dose or frequency alterations; 30% (B) to 53% (A); only dose change orders; 54% (B) to 67% (A); only frequency change orders 35% (B) to 59% (A); $p < 0.001$ for all; Mean (SD) length of stay in hospital in days: 4.5 (4.8; B) to 4.3 (4.5; A); $p < 0.009$; (without overlapping admission); 5.4 (7.4; B) to 5.3 (7.1; A); $p < 0.05$ (with overlapping admission)	Yes. More appropriate dose and frequency orders in patients with renal insufficiency from 30 to 53%	Yes, significant but small reduction in mean hospital stay in days 4.5 (B) to 4.3 (A)	No
Cordero 2004 Secondary Care United States	Before/After with control period data obtained retrospectively	12 mo B6/A6 2002 211 very-low-birth-weight infants (≤1,500 g) 111 before CPOE (B) 100 after CPOE (A)	Undetermined/Drug allergy interaction, Drug–drug interaction, Duplicate order, Corollary order alert, Dose range checking	To provide clinicians with drug–drug interaction and drug–allergy checking at the point of care	Alert/vendor-based system with graphical use interface, extensively modified to meet the needs of this pediatric population. Clinical decision support including drug–drug interaction and drug–allergy checking	Prescription dosage errors for gentamycin (prescribed dose >10% deviation from recommended dose) decrease from 13 to 0% (1/3 overdose, 2/3 underdose); 14 (B) to 0 (A) *p*-value NR	Unclear. Gentamycin dosage errors decreased from 13 to 0%. Significant?	NR	No
Dawson 2023 Secondary Care United States	Before/After	Phase 1: March 10, 2021 to January 12, 2022 Phase 2: January 13, 2022 to December 31, 2022 Phase 1: 62,147 PRN acetaminophen and 57,095 PRN ibuprofen orders Phase 2: 51,117 PRN acetaminophen orders and 47,173 PRN ibuprofen orders	Interruptive/Duplicate Order	To reduce therapeutic duplication with acetaminophen and ibuprofen orders	Alert/The alerts prompted clinicians to clarify PRN comments at order entry and there was the addition of first and second-line discrete reasons to orders	After the introduction of interruptive alerts, the number of therapeutic duplications in a 30-d period decreased by 45% from 1,485 to 818 but rose back to 1,208 in the 30-d prior to the second intervention. After discrete reasons were added to the order, therapeutic duplication decreased to 336 in the immediate 30 d and 6 mo later remained at 277	Unclear. Therapeutic duplications decreased by 45%, but no statistical test was conducted	NR	Yes
Desmedt[a] 2018 Secondary Care Belgium	Before/After	12 d Before: 6 d in September 2009 After: 6 d in September 2010 Before: 301 patients After: 314 patients	Interruptive/Dose adjustment, Dose range checking	The aim of the study was to evaluate the effectiveness of a CDSS on the appropriateness of drug dosages in patients with renal failure	Alert/The CDSS automatically compares the prescription with the recommendation for dosage adjustment, taking into account the patient's last available Glomerular Filtration Rate (GFR) and displays, in case of inappropriateness (i.e., overdosage or contraindicated drug), an alert reminding the prescriber of the patient's renal clearance and of the drug dosage recommended for this clearance. The physician can modify, or not, the prescription according to the recommendation. The prescriber was not asked for a justification	The dosage was inappropriate in 25.4 and 24.6% of prescriptions in the pre-and postimplementation periods (OR: 0.97; 95% CI: 0.72–1.29)	No	NR	No

Supplementary Appendix 2: (*Continued*)

Author<br>Year<br>Setting<br>Country	Study design/methods	Duration and participants	Type of alert/alert category	Alert objectives	Alert, reminder, or prompt and alert design	Outcomes/results	Beneficial effect on prescribing?	Beneficial effect on patient outcomes?	Can the outcome be attributed to a particular category of alert?
Duke[a]<br>2013<br>Primary Care<br>United States	RCT	6 mo<br>February 22, 2011 to August 30, 2011<br>101 intervention doctors and 102 control doctors	Interruptive/Drug–drug interaction, Drug laboratory alert	To determine whether enhancing DDI alerts with laboratory data relevant to hyperkalemia would improve alert adherence in high-risk patients	Alert/Upon activation, when any of these interactions involving ACE inhibitors, angiotensin receptor blockers, potassium-sparing diuretics, and potassium supplements associated with hyperkalemia were triggered by an intervention physician, the most recent values for potassium and creatinine within the past 12 mo were included in the DDI alert	No significant difference in alert adherence in high-risk patients between the intervention group (15.3%) and the control group (16.8%) ($p = 0.71$). Adherence in normal-risk patients was significantly lower in the intervention group (14.6%) than in the control group (18.6%; $p < 0.01$)	No	NR	No
Durieux<br>2000<br>Secondary Care<br>France	Time series analysis	84 wk<br>4 × 10 wk before/without intervention<br>Period (B)<br>3 × 10 wk periods postintervention (A)<br>4-wk washout between each period<br>1997/1999<br>1971 pts undergoing orthopedic surgery	Interruptive/Drug–condition interaction, Dose range checking	To increase the proportion of appropriate anticoagulant prescriptions ordered	Prompt/Computer system designed to encourage prescribing for venous thromboembolism prophylaxis. Prescribers are notified via a message on the computer screen suggesting the appropriate prescription and explaining the reasons	% of cases physicians complied with guidelines: 82.8% (B) to 94.9% (A) $p < 0.001$ RR of inappropriate prescribing in preintervention vs. postintervention period: 3.8 (95% CI, 2.7 to 5.4) equivalent to RRR of inappropriate prescribing: 73%; Appropriateness of prescription increased during each intervention period ($p < 0.001$). Each time the CDSS was removed, physician compliance with guidelines reverted to that observed before initiation of the intervention	Yes. The proportion of appropriate prescriptions increased significantly from 82.8 to 94.9%	NR	No

(*Continued*)

 © 2025. The Author(s).

Supplementary Appendix 2: (*Continued*)

Author<br>Year<br>Setting<br>Country	Study design/methods	Duration and participants	Type of alert/alert category	Alert objectives	Alert, reminder, or prompt and alert design	Outcomes/results	Beneficial effect on prescribing?	Beneficial effect on patient outcomes?	Can the outcome be attributed to a particular category of alert?
Field[a]<br>2009<br>Primary Care<br>United States	RCT	Duration: 12 mo<br>Intervention:<br>400 residents<br>(107,856 resident d)<br>Control:<br>433 residents<br>(106,111 resident days)	Interruptive/Dose range checking, Drug laboratory alert, Dose adjustment	To determine whether a computerized clinical decision support system providing patient-specific recommendations in real-time improves the quality of prescribing for long-term care residents with renal insufficiency	Alert/Four types of alerts were developed: (1) alerts recommending the maximum total daily dose of the medication; (2) alerts recommending maximum frequency of administration; (3) alerts recommending that the medication be avoided; and (4) alerts notifying prescribers that no creatinine clearance could be calculated for this resident because of missing serum creatinine test results or weight. Alerts were triggered when a physician used the CPOE system to initiate an order for one of the specific medications included in the CDSS for a resident with renal insufficiency. After initiating the order, the prescriber could have chosen to continue with the order, modify the dose or frequency, or cancel the order. Alerts were not provided during renewals. The underlying software system could not present alerts from which prescribers could directly submit drug orders so the alerts were solely informational	The proportions of dose alerts for which the final drug orders were appropriate were similar between the intervention and control units (RR: 0.95, 95% CI: 0.83, 1.1) for the remaining alert categories significantly higher proportions of final drug orders were appropriate in the intervention units: RR: 2.4 for maximum frequency (1.4, 4.4); 2.6 for drugs that should be avoided (1.4, 5.0); and 1.8 for alerts to acquire missing information (1.1, 3.4).<br>Overall, final drug orders were appropriate significantly more often in the intervention units—RR: 1.2 (1.0, 1.4). Final orders for drugs that should be avoided were submitted less often in the intervention units, 3.5 per 1,000 resident days compared to 5.2 per 1,000 resident days in the control units. The rate ratio was 0.68, but not significant (95% CI: 0.45, 1.0)	Yes	NR	Yes
Galanter<br>2004<br>Secondary Care<br>United States	Time series analysis	13 mo:<br>6B<br>7A<br>Total numbers of alerts or alerting situations:<br>B 821<br>A 775	Interruptive/Drug laboratory alert	To warn of potential risks from abnormal lab results:<br>–Unknown or abnormal electrolyte concentrations;<br>–Hypokalaemia and hypomagnesemia in patients receiving digoxin;<br>–Unknown digoxin concentrations	Alert/Pop-up box containing information about hypokalaemia, hypomagnesemia, no recent assessment of a digoxin level, a digoxin level >2.2 mg/dL, and concurrent use of medications known to increase digoxin level. No response required	Checking for unknown serum values at 1 h: Digoxin 6% (B) to 19% (A); Potassium 9% (B) to 57% (A); Magnesium 12% (B) to 40% (A);<br>$p < 0.01$ for all;<br>$p < 0.01$ also at 24 h;<br>Prescribed electrolyte supplementation with newly reported electrolyte abnormalities: Potassium 6% (B) to 35% (A); Magnesium 5% (B) to 49% (A);<br>$p < 0.01$ for all also significant B/A difference after 24 h	Yes. Alert enhances clinicians' awareness of electrolyte derivations and improves their prescribing of electrolyte supplements	NR	Yes

 © 2025. The Author(s).

Supplementary Appendix 2: (*Continued*)

Author<br>Year<br>Setting<br>Country	Study design/methods	Duration and participants	Type of alert/alert category	Alert objectives	Alert, reminder, or prompt and alert design	Outcomes/results	Beneficial effect on prescribing?	Beneficial effect on patient outcomes?	Can the outcome be attributed to a particular category of alert?
Galanter<br>2005<br>Secondary Care<br>United States	Before/After	18 mo<br>4B/14A<br>Alerts or alerting situations:<br>87 (B)<br>323 (A)	Interruptive/Drug laboratory alert	To reduce the use of contraindicated medications in patients with renal insufficiency	Alert/Alert when the clinician attempted to order one of the potentially contraindicated drugs for a patient whose most recent creatinine clearance estimate was less than the corresponding safe level for the drug. The clinician received a pop-up alert suggesting not to proceed with the order. In the alert message, the clinician was provided with the most recent creatinine clearance estimate, the established safe creatinine clearance estimate for each drug, and a pager number of the on-call pharmacist if the clinician had any questions regarding the alert. An example is provided in the paper.	Likelihood of a patient receiving at least one dose of contraindicated drug: 89% (B) to 47% (A); $p < 0.0001$; absolute risk reduction 42% Mostly due to cancellation of an order after reviewing alert	Yes. 42% less medication contraindicated for patients with renal insufficiency	NR	Yes
Garcia[a]<br>2012<br>Secondary Care<br>Spain	Before/After	6 mo<br>Before: April–June 2010, 360 patients<br>After: April–June 2011, 327 patients	Undetermined/Drug–drug interaction	An alert was added to the assisted electronic prescription program (EPP) and triggered by a prescription for concomitant clopidogrel and omeprazole treatment. A safer choice (pantoprazole or ranitidine) was suggested to assist health professionals	Alert/No further information is provided other than what is stated under Alert Objectives	The percentage of patients treated with a PPI fell from 68.9% to 24.7% (OR = 0.15; 95% CI: 0.11 to 0.21; $p < 0.05$); the percentage of these patients treated with ranitidine rose from (26.1–65.7%; OR = 5.43; 95% CI: 3.91–7.54; $p < 0.05$)	Yes	NR	Yes

(*Continued*)

Supplementary Appendix 2: (*Continued*)

Author<br>Year<br>Setting<br>Country	Study design/methods	Duration and participants	Type of alert/alert category	Alert objectives	Alert, reminder, or prompt and alert design	Outcomes/results	Beneficial effect on prescribing?	Beneficial effect on patient outcomes?	Can the outcome be attributed to a particular category of alert?
Hansen[a]<br>2018<br>Primary and Secondary Care<br>United States	Before/After	6 mo:<br>Before:<br>December 1, 2015 to February 28, 2016<br>8,106 patients<br>After: December 1, 2016 to February 28, 2017<br>8,464 patients	Interruptive/Drug–condition interaction	To determine if a best practice alert (BPA) will affect the percentage of oral antibiotic prescriptions for adults with acute rhinosinusitis (ARS)	Alert/A real-time best practice alert (BPA) was implemented in the electronic medical record to notify the provider of the Choosing Wisely recommendation when prescribing an antibiotic for a patient diagnosed with ARS during that visit. A diagnosis of sinusitis triggered a pop-up screen with a link to the Choosing Wisely recommendation and a prompt to remove the antibiotic prescription. Optional justification for continuing the antibiotic order, designated as "acknowledgment reasons" could be selected in the BPA response field. One of six discrete acknowledgment reasons, as developed by a physician-led clinical decision support committee, could be selected or entered as free text. The alert could be dismissed without requiring the clinician to provide justification for the antibiotic prescription	The percentage of oral antibiotics prescribed for the pre- and post-intervention groups were 94.8 and 94.3%, respectively ($p = 0.152$). Being in the BPA group was not associated with decreased odds of an antimicrobial prescription in the adjusted model ($p = 0.125$)	No	NR	Yes
Hashemi[a]<br>2021<br>Secondary Care<br>The Netherlands	Before/After	8 mo<br>Before:<br>From October 2019 through January 2020, 266 patients<br>After:<br>From April 2020 through July 2020, 238 patients	Passive/Dose range checking, Dose adjustment, Drug laboratory alert	This study aimed to assess the effect of a CDSS on protocol deviation (as a measure of prescription error) types and frequency in a pediatric intensive care unit (PICU)	lert/The CDSS automatically checked and displayed dosing limits, calculated medication doses, and frequency of administration. To perform the check and calculations, four components of the prescription are needed in the electronic prescribing system, three of which must be entered by the physician: 1) the generic drug, 2) the route of administration, and 3) the indication for prescribing the drug. The fourth component, patient category (e.g., age, gestational age, weight, and body surface area), is directly taken from the patient data management system (PDMS), so do not have to be manually entered for each prescription. After these components are known, the CDSS will immediately display the specific dosing information, including recommended dose, upper and lower limits, and a list of applicable administration frequencies	A significant reduction was observed in the total number of protocol deviations per 100 prescriptions, from 0.89% pre-CDSS to 0.49% post-CDSS ($p = 0.02$). The number of protocol deviations outside the recommended dosing limits significantly decreased from 0.74% pre-CDSS to 0.39% post-CDSS ($p = 0.03$)	Yes	NR	No

 © 2025. The Author(s).

Supplementary Appendix 2: (*Continued*)

Author Year Setting Country	Study design/methods	Duration and participants	Type of alert/alert category	Alert objectives	Alert, reminder, or prompt and alert design	Outcomes/results	Beneficial effect on prescribing?	Beneficial effect on patient outcomes?	Can the outcome be attributed to a particular category of alert?
Hulgan<br>2004<br>Secondary Care<br>United States	Time series analysis	104 wk:<br>52B<br>52A<br>15,194 quinolone orders	Interruptive/ Intravenous to oral conversion	To increase the proportion of oral quinolone antibiotic orders by decreasing unnecessary IV orders	Prompt/Screenshots suggesting to prescribe oral quinolone if an order for IV quinolone was initiated	Oral quinolone orders 4,202/7,571 = 55.5% (B); 4,760/7,623 = 62.4% (A); 558 more oral orders = increase by 13.3%. In the time series analysis, increased proportion of quinolone orders per week by 5.6% (95% CI 2.8 to 8.4%, $p < 0.001$). MDs placed 77% of all quinolone orders. Sub-analysis found that among MDs there was an increase in the proportion of oral quinolones per week of 6.0% (95% CI 2.8 to 9.3%, $p < 0.001$). The most common reason for overriding the CDSS was "patient unable to take oral medications"	Yes. Increases proportion of oral quinolone orders	NR	Yes
Jajosky[a]<br>2019<br>Secondary Care<br>United States	Before/After	6 mo<br>Before: January 20, 2013, to April 20, 2013<br>37 patients<br>After: April 21 2013 to June 21, 2013<br>39 patients	Interruptive/Drug–condition interaction, Drug–drug interaction, Formulary alert	To help decrease the risk of epidural hematoma, the authors developed an alert in the electronic medical record to assist providers with adherence to published guidelines addressing neuraxial analgesia and anticoagulation	Alert/The best practice alert (BPA) would appear when a physician initiates or modifies an anticoagulant order for a patient with a documented epidural. The BPA would require administrators to select an "Accept" or "Cancel" button before proceeding to the medication order screen. Both the Accept and Cancel buttons would make the BPA disappear and allow the ordering provider to order the anticoagulant at any dose without a further barrier. For patients with epidural catheters, the BPA would be expected to fire anywhere between 1 and 5 times. Documentation of an epidural catheter in the day-of-care flow sheet by the bedside nurse (usually entered at the time of placement or shortly after epidural placement) was used to trigger the BPA. The BPA would not become active if the epidural catheter was not documented in the flow sheet	Patients with epidural catheters placed after the BPA observed a 61% decrease in the expected number of days of exposure to inappropriate doses of anticoagulation vs. patients treated before the implementation of the alert. A Poisson regression revealed that alert status (before vs. after) significantly ($p < 0.05$) predicted inappropriate anticoagulant use when age, anticoagulant type, and indication (rib fractures vs. postoperative pain) were also included in the model	Yes	NR	No

(*Continued*)

 © 2025. The Author(s).

Supplementary Appendix 2: (*Continued*)

Author<br>Year<br>Setting<br>Country	Study design/methods	Duration and participants	Type of alert/alert category	Alert objectives	Alert, reminder, or prompt and alert design	Outcomes/results	Beneficial effect on prescribing?	Beneficial effect on patient outcomes?	Can the outcome be attributed to a particular category of alert?
Judge<br>2006<br>Secondary<br>United States	RCT	1 y<br>2002/2003<br>4,282 alerts from 47,997 medication orders relating to 3,726 resident mo of observation time	Interruptive/Drug–drug interaction; Drug-laboratory alert; Dose range checking; Corollary order alert	To improve medication safety in the long-term care setting by influencing prescribing behavior	Alert/13 categories of alerts:<br>–Related to CNS<br>–Constipation side effects<br>–Warfarin related<br>–Renal insufficiency<br>–Hypokalaemia<br>–Hyperkalemia<br>–Drug dose<br>–Hyperglycemia<br>–Anticholinergic SE<br>–Antiplatelet effect<br>–Drug interaction<br>–Phenytoin<br>–Low TSH level<br>Examples of alert messages are shown in the paper	Appropriate prescribing: 28% (C) to 31% (I); RR: 1.11; (95% CI: 1.00–1.22). Alerts were generated in 19.6% of the medication orders. Alert categories most often triggered were related to risks of central nervous system side-effects such as over-sedation (20%), alerts for risk of drug-associated constipation (13%), renal insufficiency/ electrolyte imbalance (12%),<br>and warfarin orders (12%)	No	NR	Yes
Kang[a]<br>2018<br>Secondary Care<br>United States	Before/After	9 mo<br>Before:<br>November 1, 2013 to January 31, 2014,<br>2,881 medication orders<br>After:<br>November 1, 2014 to January 31, 2015,<br>2,700 medication orders	Interruptive/Formulary alert, Dose adjustment, Dose range checking	The study evaluated the effectiveness of using clinical decision support with alerts to reduce medication error rates	Alert/Alerts for 3 commonly used classes of medications: angiotensin-converting enzyme inhibitors (ACEIs), angiotensin II receptor blockers (ARBs), and 3-hydroxy-3-methylglutaryl coenzyme A reductase inhibitors (HMG-CoA RIs). If a nonformulary medication within the selected medication classes was ordered, a pop-up prescriber alternative alert proposed a therapeutic equivalent along with appropriate dosing conversion ratios	Formulary adherence improved from 78.3% to 97.6% ($p < 0.001$). Inappropriate dosing conversions were reduced from 51.6% to 37.2% (NS) and inappropriate discharge medications were reduced from 64.5% to 16.3% (NS)	Yes	NR	Yes
Keohane[a]<br>2016<br>Primary Care<br>Ireland	Before/After	3 mo before, 3 mo after. 158 patients with impaired renal function and an eGFR of 60 mL/min or less (same participants in the before and after phases)	Interruptive/Drug laboratory alert, Drug–condition interaction	To reduce inappropriate nonsteroidal anti-inflammatory prescribing to those with CKD	Alert/When an NSAID was prescribed, a message alerted the doctor to the relative contra-indication and included the patient's most recent eGFR and CKD stage. It was possible to over-ride this message and prescribe the NSAID, but this decision could only be made after considering the patients' renal function	A significant 75 percent reduction in the total nonsteroidal anti-inflammatories prescribed and a 90 percent reduction in repeat nonsteroidal anti-inflammatory prescriptions in those with CKD. No statistical tests were run	Yes. No statistical tests run	NR	No

 © 2025. The Author(s).

Supplementary Appendix 2: (*Continued*)

Author<br>Year<br>Setting<br>Country	Study design/methods	Duration and participants	Type of alert/alert category	Alert objectives	Alert, reminder, or prompt and alert design	Outcomes/results	Beneficial effect on prescribing?	Beneficial effect on patient outcomes?	Can the outcome be attributed to a particular category of alert?
Khalil[a]<br>2021<br>Secondary Care<br>Australia	Before/After	4 mo<br>Before: January 2019 to March 2019<br>After: January 2020 to March 2020<br>100 patients before (representing 67 prescribers) and 100 patients after (representing 58 prescribers; not the same patients in the before and after phases)	Undetermined/Dose range checking	To improve the appropriateness of NOAC prescribing	Alert/The intervention focused on patient-specific alerts based on patients' physiological parameters such as age, renal function, weight, and drug interactions to improve the appropriateness of NOAC prescribing	Improved appropriateness of NOAC prescribing from 48 to 91%, $p < 0.05$. The implementation of NOAC alerts reduced inappropriately prescribed high doses of NOACs (39% vs. 2%, $p = 0.00261$) and inappropriately prescribed low doses of NOACs ($p = 0.00245$), but did not prevent the incorrect type (as suited to the patient comorbidities) of NOAC being prescribed (4% vs. 1%, $p = 0.71657$)	Yes	Not significant, the total number of reported hospital-acquired complications that are associated with inappropriate NOAC prescribing was reduced by 36% in the postintervention phase (from 29 to 22 (RR $= 0.7454$, $p = 0.2986$)	Yes
Kostourou<br>2023<br>Secondary Care<br>Greece	Before/After	June 14, 2014 to September 30, 2017<br>400 patients in preintervention phase and 680 in postintervention phase	Interruptive/Dose adjustment	To increase adherence to hospital guidelines for perioperative antimicrobial prophylaxis	Reminder/The prescribing physician had to select whether the antimicrobial would be therapeutic or prophylactic. When the physician selected "prophylactic" a reminder with a link to the PAP Hospital Guidelines appeared. The intervention was not restrictive regarding the choice of antibiotic; however, the amount of antimicrobial dispensed was adjusted to the appropriate duration, that is, 48 h for cardiac surgery	Adherence to the appropriate duration of PAP increased significantly after the intervention [pre 4.0% (16/399) vs. post 15.4% (105/680), $p < 0.001$]	Yes	No, the rate of serious surgical infections increased significantly during the study (pre 2.8% (11/400) vs. post 5.9% (40/680) $p < 0.019$). Documented postoperative infections other than SSIs also increased significantly [pre 6.0% (24/400) vs. post 10.3% (70/680), chi-square $p < 0.016$]. Length of index hospitalization decreased marginally after the intervention [median, IQR 10 (9–13) vs. 10 (8–13), $p = 0.001$, while In-hospital mortality was not affected by the intervention [PRE 3.8% (15/400) vs. post 4.8% (33/680), $p = 0.39$]	Yes

(*Continued*)

 © 2025. The Author(s).

Supplementary Appendix 2: (*Continued*)

Author<br>Year<br>Setting<br>Country	Study design/methods	Duration and participants	Type of alert/alert category	Alert objectives	Alert, reminder, or prompt and alert design	Outcomes/results	Beneficial effect on prescribing?	Beneficial effect on patient outcomes?	Can the outcome be attributed to a particular category of alert?
Lee[a]<br>2014<br>Secondary Care<br>South Korea	Before/After	12 mo<br>Before:<br>From January to June 2009, 176,353 prescriptions<br>After:<br>From January to June 2010, 181,064 prescriptions	Interruptive/Corollary order alert, Dose range checking, Dose adjustment, Formulary alert	To evaluate the impact of a high-alert medication clinical decision support system on point-of-order entry errors	Alert/On the CPOE window, a warning icon is displayed just in front of the drug code. When the mouse arrow hovers above the icon, a "frequent errors" or "key warning" message is displayed. Moreover, when the icon is double-clicked, the clinicians obtain HTML documents that contain clinical information. The system also produced a pop-up when erroneous orders were prescribed and provided the clinician with order recommendations	The dilution fluid was omitted from regular insulin orders in 31.1% before deployment, but after deployment, there were no such cases ($p < 0.001$). With regard to potassium chloride, there were 5.6% dilution fluid omissions before deployment, but no such cases occurred after deployment ($p < 0.01$). Administration route changes changed significantly after deployment for all drugs ($p < 0.01$). The doses that exceeded the maximum also declined significantly after deployment ($p < 0.01$)	Yes	NR	No
Lester[a]<br>2015<br>Secondary Care<br>United States	Before/After	1 y<br>Before: spring of 2010, 7,502 patients<br>After: the spring of 2011–2013, 29,613 patients	Interruptive/Formulary alert	To improve prescribing among older hospitalized patients by adding alerts to the CPOE system for potentially inappropriate medications	Alert/Informational alerts were integrated into the CPOE system for selected high-risk medications: diphenhydramine, metoclopramide, and all antipsychotics. These pop-up alerts contained links to articles relevant to the alert. The alerts required the user to click ''noted'' and then the user either canceled their order or continued with prescribing the medication. The alert did not require an explanation for the use of the medication	There were significant reductions in prescription rates prealert vs. postalert for diphenhydramine ($p < 0.01$) and metoclopramide ($p < 0.01$). There was no significant decrease in prescription rates for antipsychotics ($p = 0.80$)	Yes	NR	Yes

 © 2025. The Author(s).

Supplementary Appendix 2: (*Continued*)

Author<br>Year<br>Setting<br>Country	Study design/methods	Duration and participants	Type of alert/alert category	Alert objectives	Alert, reminder, or prompt and alert design	Outcomes/results	Beneficial effect on prescribing?	Beneficial effect on patient outcomes?	Can the outcome be attributed to a particular category of alert?
Li[a]<br>2022<br>Primary Care<br>Taiwan	Time series analysis	January 1, 2015, and December 31, 2018. This study was based partly on data from the Big Data Center, TPEVGH which serves >2.5 million outpatient visits for 1.1 million outpatients yearly in northern Taiwan. On average, ~25,000 drug prescriptions were generated daily for ambulatory patients	Interruptive/Dose adjustment, Dose range checking, Drug–drug interaction, Drug laboratory alert, Duplicate order	Evaluated the short- and long-term effects of clinical decision support systems (CDSS) on inappropriate prescriptions of glucose-lowering agents for patients with renal insufficiency in an ambulatory care setting	Alert/The CDSS for dose suggestions in patients with renal insufficiency, the Renal Function Alert Plus Individualized Dose Suggestion System (RAS-Plus), was used on January 7, 2016. RAS-Plus, designed by a team of pharmacists, physicians, and computer programmers, was integrated into CPOE and linked to the laboratory database in the hospital information system. After the expert panel reviewed all the drugs in the hospital, 460 drugs that needed dose adjustments based on the renal function of the patients were added to the RAS–Plus knowledge database. The recommended dosage of these drugs was established according to different levels of renal impairment in the knowledge database. After the CDSS was implemented, it immediately checked and provided alerts whenever a physician prescribed a medication for a patient. When the physician prescribes a medication that requires dose adjustments on the basis of renal function (e.g., sitagliptin, which is a drug with a definite dose recommendation) to a patient aged >18 y and an estimated glomerular filtration rate (eGFR) < 50 mL/min/1.73 m^2 or on dialysis, RAS-Plus verifies the dose, dosing frequency, and the daily dose of the prescriptions. In cases in which the dose or frequency exceeds the system setting, RAS-Plus then immediately calculates the appropriate dosage according to the patient's renal function individually and provides the recommended dose and frequency to the physicians in a pop-up window on the CPOE screen. In cases in which a medication without a definitive dose recommendation according to the patient's renal function, such as metformin, was prescribed to patients with renal failure, RAS-Plus then only provides context alerts. If the patient is aged >65 y and there were no renal function data in the previous 2 y, RAS-Plus will issue an alert that reminds physicians to check the patient's renal function. Any alert provided by RAS–Plus and whether the physicians accepted them were logged	In the short-term analysis, the baseline inappropriate rate for overall medications was estimated to range from 30.54% in the first month to 27.06% in month 12. The predicted inappropriate rate 12 mo after implementation was 19.35%, corresponding to an estimated 28.49% [(27.06–19.35)/27.06] decrease in inappropriate rate. However, after long-term analysis, the predicted inappropriate rate at the end of the study (36 mo after implementation) was 18.02%. A total of 27,189 alerts were generated and 628 were accepted during the study period. Thus, after short- and long-term analysis, the overall acceptance rate was 3.06 and 2.31%, respectively	Yes	NR	No

(*Continued*)

 © 2025. The Author(s).

Supplementary Appendix 2: (*Continued*)

Author<br>Year<br>Setting<br>Country	Study design/methods	Duration and participants	Type of alert/alert category	Alert objectives	Alert, reminder, or prompt and alert design	Outcomes/results	Beneficial effect on prescribing?	Beneficial effect on patient outcomes?	Can the outcome be attributed to a particular category of alert?
Lilih[a]<br>2017<br>Secondary Care<br>The Netherlands	Before/After	3 mo before (May 1, 2014–August 1, 2014) and 3 mo after implementation (December 1, 2014–March 1, 2015).Included patients who were at risk for developing gastrointestinal bleeding and should be prescribed gastrointestinal prophylaxis according to the Dutch guideline. All inpatients and outpatients in the Hoofddorp site of the Spaarne Gasthuis who had an indication for gastrointestinal prophylaxis were included. This study was performed in the Hoofddorp site of the Spaarne Gasthuis, a site with 455 beds	Interruptive/Corollary order alert, Dose adjustment, Dose range checking, Drug allergy interaction, Drug–condition interaction, Drug–drug interaction	The primary objective was to determine whether the implementation resulted in improved compliance with this guideline for gastrointestinal prophylaxis. A secondary objective was to determine whether implementation resulted in a reduction in the number of drug safety alerts	Alert/In the clinical decision support system alert, a single pop-up is shown with a short description and the possibility to order pantoprazole 40 mg once daily by clicking once. Pantoprazole 40 mg once daily is mentioned in the Dutch guideline as a suitable drug and dose for gastrointestinal prophylaxis. In the case of children, a similar alert is shown which advises starting with gastrointestinal prophylaxis, without recommending an order. If the prescriber does not order gastrointestinal prophylaxis, a reason should be selected from the menu in the alert. The way physicians can override this alert without ordering gastrointestinal prophylaxis is similar to overriding the classical medication surveillance alert. Prescribers were informed about the introduction of this clinical decision support system in the newsletter that accompanies each update of the hospital information system. For inpatient orders, a list of patients with an indication for gastrointestinal prophylaxis but without a prescription is reviewed on a daily basis by a pharmacist, and prescribers are contacted by telephone if needed. The alert in the clinical decision support system is based on an algorithm, in which patient characteristics are taken into account for the generation of patient-specific drug safety alerts. The clinical decision support for gastrointestinal prophylaxis includes the use of risk medication(s), the use of gastrointestinal prophylaxis, and age as a risk factor. Other risk factors, such as a diagnosis of diabetes, were not incorporated, because this information is not unequivocally documented in the hospital information system. The algorithm does not generate an alert if the patient has already been prescribed gastrointestinal prophylaxis, thereby further limiting the pop-up of irrelevant alerts	Before implementation in 84.0% of the included 2,064 prescriptions, gastrointestinal prophylaxis was co-prescribed. After implementation, this percentage increased to 94.5% of the 2,269 prescriptions ($p < 0.001$). The number of drug safety alerts decreased by 78.2% from 980 to 217 alerts	Yes	NR	No

 © 2025. The Author(s).

Supplementary Appendix 2: *(Continued)*

Author Year Setting Country	Study design/methods	Duration and participants	Type of alert/alert category	Alert objectives	Alert, reminder, or prompt and alert design	Outcomes/results	Beneficial effect on prescribing?	Beneficial effect on patient outcomes?	Can the outcome be attributed to a particular category of alert?
Mahoney[b] 2007 Secondary Care United States	Before/After	Before was variable in terms of dates After: January 1, 2005, through March 31, 2006, and January 1 2006 through June 30, 2006. 1,452,346 inpatient medication orders in the preimplementation period and 1,390,789 in the postimplementation period	Interruptive/Drug allergy interaction; Dose range checking; Duplicate order, Dose adjustment	To reduce medication errors and improve therapeutic drug monitoring in patients with renal insufficiency and in patients receiving drugs with narrow therapeutic ranges	Alert/The CPOE system provides the prescriber with alerts such as the detection of an allergy to the prescribed medication and therapeutic duplication. Drug-specific alerts were also programmed into the system to provide information such as dosage adjustment or monitoring requirements during order entry. The upgraded pharmacy system had the ability to program order-entry alerts on the basis of laboratory result data and the prescribed medication, as well as the availability of maximum dosage alerts	Drug allergy; 883 errors; before; 109 errors after; OR 0.14; $p < 0.001$; Excessive dose; 1,341 errors before; 871 errors after; OR 0.68; $p < 0.001$; Therapeutic duplication; before; 665 errors; after; 584 errors; OR 0.92; $p = 0.127$; Number of pharmacist-initiated dosage adjustments for renal insufficiency increased from 446 to 935 (OR: 2.13 $p < 0.001$); Rate of pharmacist initiated dosage adjustments for serum drug concentrations outside of the therapeutic range increased from 42 to 258 (OR, 6.24 $p < 0.001$)	Yes	NR	Yes
Malte[a] 2018 Secondary Care United States	Time series analysis	Evaluated the implementation of an advanced medication alert designed to identify Veterans with known high-risk conditions (SUD, suicide risk, sleep apnea, age 65 and above) who were co-prescribed opioids and benzodiazepines. The alert was implemented in 2014 at 1 multisite VA healthcare system and evaluated over a 1-y period	Interruptive/Drug–condition interaction, Drug–drug interaction, Formulary alert	Evaluated the effectiveness of a medication alert designed to reduce opioid and benzodiazepine co-prescribing among Veterans with known high-risk conditions (substance use, sleep apnea, suicide risk, age 65 and above) at 1 Veterans Affairs (VA) healthcare system	Alert/Three criteria were required to activate the alert: (1) the prescriber ordered an outpatient benzodiazepine/opioid medication; (2) the patient had an active VA or documented non-VA prescription for the other medication class; and (3) the patient had a risk condition documented in the EMR in the past 12 mo. Risk conditions activating the alert were: SUD diagnosis or severe alcohol misuse per the Alcohol Use Disorders Identification Test-Consumption (AUDIT-C) 33; suicide-risk, defined as a patient record flag,34 suicide attempt, or inpatient MH admission35; sleep apnea; and age 65 and above. The alert activated at the point of prescribing within the EMR,36 synthesizing patients' current benzodiazepine and/or opioid prescriptions and risk factors that activated the alert. Relevant EMR details were provided (e.g., substance use or sleep apnea diagnosis date), enabling prescribers to review information. The alert was not activated if no targeted risk factors were present. Providers could ignore or override the alert without justification	Proportions of patients with concurrent prescriptions decreased significantly postalert launch among substance use [adjusted odds ratio (aOR) = 0.97; 95% CI = 0.96–0.99; 12-mo decrease = 25.0%], sleep apnea (AOR = 0.97, 95% CI = 0.95–0.98, 12-mo; Decrease = 38.5%), and suicide-risk (AOR = 0.94, 95% CI = 0.91–0.98, 12-mo decrease = 61.5%) cohorts at the alert site. Decreases in co-prescribing were significantly different from the comparison site among suicide risk (AOR = 0.92, 95% CI = 0.86–0.97) and sleep apnea (AOR = 0.98, 95% CI = 0.96–1.00) cohorts. Significant decreases in benzodiazepine prescribing trends were observed at the alert site only	Yes	NR	No

(Continued)

 © 2025. The Author(s).

Supplementary Appendix 2: (*Continued*)

Author<br>Year<br>Setting<br>Country	Study design/methods	Duration and participants	Type of alert/alert category	Alert objectives	Alert, reminder, or prompt and alert design	Outcomes/results	Beneficial effect on prescribing?	Beneficial effect on patient outcomes?	Can the outcome be attributed to a particular category of alert?
Martens<br>2007<br>Primary Care<br>The Netherlands	Cluster RCT	12 mo<br>7 GP practices (25 GPs) received reminders on antibiotics and asthma/COPD prescriptions 7 GP practices (28 GPs) received reminders on cholesterol prescriptions	Interruptive/Drug–condition interaction	To influence drug-prescribing behavior in general practice: To encourage/discourage certain drugs according to clinical guidelines<br>1. Antibiotic prescribing (encourage certain Abs in acne but discourage in URTI)<br>2. Asthma/COPD medication<br>3. Statins	Reminder/Automated feedback system. Screenshot. Reminders on specific indication, alternative type of drug, alternative drug administration, other doses, other duration, not to prescribe anything or to specialist	Prescriptions per 1,000 patients per GP: Discourage antibiotics 39.7 (C) to 28.2 (I); $p = 0.2$; Encourage antibiotics 20.5 (C) to 20.7 (I) $p = \text{NS}$; Discourage asthma/COPD meds 2.2 (C) to 1.1 (I); $p = 0.1$; Encourage asthma/COPD meds 7.7 (C) to 5.9 (I); $p =$ NS; Advise Statin treatment 1.2 (C) to 1.0 (I); $p = \text{NS}$	No. Reminders to encourage/discourage certain prescribing patterns did not influence prescribing behavior significantly. Study underpowered	NR	Yes
Matsamura[a]<br>2009<br>Secondary Care<br>Japan	Before/After	2 mo.<br>484 before the intervention. 486 after the intervention	Interruptive/Drug laboratory alert, Drug–condition interaction, Dose range checking	Developed an alert system for evaluating renal function and checking doses of medication according to the patient's renal function. In addition, developed functions of extracting target problems from the raw data and verifying if contraindicated medication has been prescribed	Alert/This system scrutinizes data handled in the CPOE system. It picks up the data needed to ascertain problems and the data of medication entered from the order entry system. First made an alert system for renal dysfunction. Creatinine clearance (Ccr) of a patient was calculated by the estimate equation of Cockcroft and Gault. If a patient's data fulfills the condition of impaired renal function, the alert message is sent to the database. The alert system also checks the dosage of each medication according to a patient's renal function. When the dosage is over-prescribed, an alert is sent. Next, made an alert system targeting contraindications for liver diseases, renal diseases, and diabetes mellitus. The criteria for these problems were set in the knowledge base. If a patient's data meets the criteria, that fact is stored in the problem database. The system also keeps a prescription check master and checks whether the patient has a problem which is a contraindication of the prescribed medication. If a problem exists, an alert is sent to the alert message database. The alert-presenting module is a web system. After accepting the patients' ID indicated by a user, the system searches the alerts concerning the patients from the database and constructs pages presenting the alert message	Compared to the period during which the contraindicated medication was prescribed before and after the alert system was put into operation. Of the patients with renal dysfunction who were prescribed the contraindicated medication, 24% had their medication discontinued before the alert system was put into operation. In contrast, the rate significantly increased to 54% after the alert system began to function ($p = 0.01$)	Yes	NR	No

 © 2025. The Author(s).

Supplementary Appendix 2: (*Continued*)

Author<br>Year<br>Setting<br>Country	Study design/methods	Duration and participants	Type of alert/alert category	Alert objectives	Alert, reminder, or prompt and alert design	Outcomes/results	Beneficial effect on prescribing?	Beneficial effect on patient outcomes?	Can the outcome be attributed to a particular category of alert?
Mazzaglia[a]<br>2016<br>Primary Care<br>Italy	RCT	Duration: 2 y<br>115 GPs in the intervention and 115 GPs in the control group	Interruptive/Drug–drug interaction, Drug laboratory alert, Drug–condition interaction	Improve the pharmacological management of high-risk cardiovascular patients (those with diabetes, myocardial infarction, and stroke). Whether the CDSS may increase the prevalence of preventive therapies, as recommended in the treatment guidelines, and reduce the number of days of exposure to potentially interacting drugs among high-risk cardiovascular patients	Alert/Messages displayed: 'Be aware! There might be a possible clinically relevant drug interaction between DRUG1 [name of the prescribed cardiovascular drug] that you are prescribing and DRUG2 [name of the drug prescribed within 30 d prior to prescription of DRUG1] which was prescribed in the last 30 d. The prompt also described the potential clinical event and suggested how to deal with this potential interaction. 'Be aware! The patient might be at high risk of developing cardiovascular diseases or further recurrences. Guidelines recommend the use of [name of not prescribed drug class according to the activated diagnosis] in such patient; BP should be < [values recommended according to the activated diagnosis] and LDL cholesterol <100 mg/dL	Significant increase in the proportion of patients with diabetes prescribed antiplatelet drugs (intervention: +2.7% vs. control: +-0.15%; $p < 0.001$) or lipid-lowering drugs (+4.2% vs. +2.8%; $p = 0.001$), no significant effects on patients with myocardial infarction or stroke. A statistically significant decrease in days of potential interactions only among patients with stroke (−1.2 vs. −0.5 d/person-year; $p = 0.001$)	Yes	NR	No
McCoy[a]<br>2010<br>Secondary Care<br>United States	Time series analysis	Before:<br>October 10, 2016 to August 10, 2007<br>After:<br>October 14, 2007 to May 16, 2008<br>1,598 adult inpatients with a minimum 0.5 mg/dL increase in serum creatinine over 48 h following an order for at least one of 122 nephrotoxic or renally cleared medications	Passive and Interruptive/Drug laboratory alert (passive and interruptive), Dose range checking (interruptive)	Evaluated the use of a computerized provider order entry intervention to improve medication management during acute kidney injury (AKI)	Alert/Passive, noninteractive warnings about increasing serum creatinine appeared within the computerized provider order entry interface and on printed rounding reports. For contraindicated or high-toxicity medications that should be avoided or adjusted, an interruptive alert within the system asks providers to modify or discontinue the targeted orders, mark the current dosing as correct and to remain unchanged, or defer the alert to reappear in the next session	The modification or discontinuation rate per 100 events for medications included in the interruptive alert within 24 h of increasing creatinine improved from 35.2 preintervention to 52.6 postintervention ($p < 0.001$); orders were modified or discontinued more quickly ($p < 0.001$). During the postintervention period, providers initially deferred 78.1% of interruptive alerts, although 54% of these were eventually modified or discontinued prior to patient death, discharge, or transfer. The response to passive alerts about medications requiring review did not significantly change when compared to baseline	Yes	NR	No

(*Continued*)

Supplementary Appendix 2: (*Continued*)

Author<br>Year<br>Setting<br>Country	Study design/methods	Duration and participants	Type of alert/alert category	Alert objectives	Alert, reminder, or prompt and alert design	Outcomes/results	Beneficial effect on prescribing?	Beneficial effect on patient outcomes?	Can the outcome be attributed to a particular category of alert?
Nelson[a]<br>2022<br>Secondary Care<br>United States	Before/After	January 2019 to April 2021 at a large academic health system in the Southeast.<br>Before:<br>January 2019 to January 2020<br>After:<br>January 2020 to April 2021<br>Before:<br>20,334 patients<br>After:<br>22,772 patients	Interruptive/Corollary order alert, Dose range checking, Drug–condition interaction, Drug–drug interaction	To determine whether a targeted clinical decision support alert could increase the rate of naloxone co-prescribing	Alert/Developed a targeted point of care decision support notification in the electronic health record to suggest ordering naloxone for patients who have a high risk of opioid overdose based on a high morphine equivalent daily dose (MEDD) ≥90 mg, concomitant benzodiazepine prescription, or a history of opioid use disorder or opioid overdose	The baseline naloxone co-prescribing rate in 2019 was 0.28 (95% CI: 0.24–0.31) naloxone prescriptions per 100 opioid prescriptions. After alert implementation, the naloxone co-prescribing rate increased to 4.51 (95% CI, 4.33–4.68) naloxone prescriptions per 100 opioid prescriptions ($p < 0.001$). The adjusted odds of naloxone co-prescribing after alert implementation were approximately 28 times those during the baseline period (95% CI, 15–52)	Yes	NR	No
Pell[a]<br>2014 Secondary Care<br>United States	Before/After	Before: 8 mo from December 2011 to July 2012<br>After:<br>8 mo from September 2012 to April 2013<br>66 alerts before, 87 alerts after were generated for IV haloperidol in patients with a QTc >500 ms	Interruptive/Drug–condition interaction	The primary objective was to decrease unsafe use of IV haloperidol in patients with prolonged QTc	Alert/A best practice advisory was fired at the initiation of an order for IV haloperidol if a patient's last ECG demonstrated a QTc interval that was >500 ms, as determined by the automated ECG machine analysis	A decrease in the rate of inappropriate haloperidol prescription from an average of 4.1/mo preintervention to 1.5/mo postintervention ($p = 0.00025$). The proportion of patients administered inappropriate haloperidol dropped from 50% (SD 15%) to 14% (SD 12%)	Yes	NR	Yes
Peterson<br>2005<br>Secondary Care<br>United States	Time series analysis	24 wk:<br>4 × 6 wk<br>(C-I-C-I)<br>3,718 patients<br>3,908 prescriptions	Interruptive/Dose adjustment, Dose range checking, Formulary alert, Drug laboratory alert	To reduce the prescribing of psychotropic medication in the elderly (benzodiazepine, neuroleptics, and opiates)	Alert/dialog box prompts presenting recommendations and alternatives	Results in the percentage of total orders; Doses reduction in: 18.6% (B) to 29.3% (A); $p < 0.001$; Prescribing of nonrecommended drugs: 10.8% (B) to 7.6% (A); $p < 0.001$; Number of falls per 100 patient days: 0.64 (B)–0.28 (A); $p < 0.001$; Mental state: Nonsignificant changes	Yes. Improved prescribing of psychotropics in the elderly: lower dose; fewer nonrecommended drugs	Yes, less falls as a result of altered prescribing patterns ($p = 0.01$). No, no change in the mental state	No

Supplementary Appendix 2: (*Continued*)

Author<br>Year<br>Setting<br>Country	Study design/methods	Duration and participants	Type of alert/alert category	Alert objectives	Alert, reminder, or prompt and alert design	Outcomes/results	Beneficial effect on prescribing?	Beneficial effect on patient outcomes?	Can the outcome be attributed to a particular category of alert?
Prasert[a]<br>2018<br>Secondary Care<br>Thailand	Before/After	Two phases with a duration of 12 mo each: pre-CDSS implementation (October 2015-March 2016) and post-CDSS implementation (October 2016-March 2017). A total of 11 258 patients were included in the period prior to CDSS implementation, and 11 915 patients were included in post-CDSS implementation	Interruptive/Drug–condition interaction	A computerized decision support system (CDSS) for the detection of potentially inappropriate medications (PIM) is a novel alert system in Thailand for reducing PIM prescriptions. The aim of this study was to evaluate the effect of a CDSS on PIM prescriptions for elderly patients in Thai community hospitals	Alert/The alert system uses the Lists of Risk Drugs for Thai Elderly (LRDTE) criteria to automatically check for PIM prescriptions and presents three medication severity levels: a mild PIM alert indicates that the drug may be used for short term or with intensive monitoring; moderate PIM alert indicates that it is best to avoid drug usage and consider alternative choices; and severe PIM alert indicates that drug usage is not recommended and offers no benefit to the patient. All medicines were ordered by physicians through the hospital software. When the alert system identifies a PIM, a message describing the possible consequences and suggesting alternative therapies is shown on the physician's computer screen. The physician could then decide to accept or override the alert message	The overall prevalence of PIM prescriptions post-CDSS implementation significantly decreased from 87.7% to 74.4%. The severity of mild and moderate PIMs was significantly reduced from 71.9 to 49.0% and from 64.5 to 48.7%, respectively. All hospitals had only one severe PIM, which was hyoscine. It was reduced from 4.7 to 1.5%, but the change was not significant ($p = 0.74$). The proportion of frequently prescribed PIMs in all PIM levels was significantly decreased, regardless of existing alternative medications	Yes	NR	Yes
Rabbani[a]<br>2023<br>Secondary Care<br>United States	RCT	December 15, 2021, and June 30, 2022. Over the 28 wk of observation,143 providers (75 in the intervention group,68 in the control group) triggered 695 alerts (345 interruptive alerts and 350 silent alerts) across 623 encounters (320 in the intervention group, 303 in the control group)	Interruptive/Formulary alert	Hypothesized that an interruptive alert may be a more scalable solution by increasing provider awareness of new HER functionality and prompting providers to update user-level preference lists on their own. Two versions of an interruptive HER alert—one "visible" and one "silent"—were created that triggered when a provider attempted to sign a discharge medication using the free-text method	Alert/Providers whose user ID ended in an even number received the visible alert, which included educational information on the safety limitations of free-text prescriptions and instructions detailing preferred prescribing methods. Two options are provided to the user:(1)acknowledge and override to submit the original prescription or (2) cancel and return to the prescription ordering activity. Providers whose user ID ended in an odd number and attempted to sign a free-text prescription received no visual feedback. Instead, a silent alert was logged in the background without an interruption to the user's workflow	Over the 28-wk study period,143 providers triggered 695 alerts (345 visible and 350 silent). The proportions of encounters with free-text prescriptions were. 83% (266/320) and 90% (273/303) in the intervention and control groups, respectively ($p = 0.01$). For the active alert, the median time to action was 31 s. Alert trigger rates between groups were similar over time. Ibuprofen, oxycodone, steroid tapers, and oncology-related prescriptions accounted for most free-text prescriptions. A Majority of these prescriptions originated from user preference lists	Yes	NR	Yes

(*Continued*)

Supplementary Appendix 2: *(Continued)*

Author<br>Year<br>Setting<br>Country	Study design/methods	Duration and participants	Type of alert/alert category	Alert objectives	Alert, reminder, or prompt and alert design	Outcomes/results	Beneficial effect on prescribing?	Beneficial effect on patient outcomes?	Can the outcome be attributed to a particular category of alert?
Rind<br>1994<br>Secondary Care<br>United States	Time series analysis	18 mo:<br>9 (B)<br>9 (A)<br>20,228 Admissions<br>14,130 patients	Passive/Drug laboratory alert	Flagging up raising creatinine levels to aim at prescribing alterations	Alert/Pop-up of information on raising creatinine levels in patients receiving nephrotoxic or renally excreted drugs. Medication and creatinine trends are clearly stated. The prescriber's response is optional	Meantime (in h) from the event until dose adjustment or drug discontinuation: 97.5 (B) to 75.9 (A); 21.1 h sooner; $p < 0.0001$; Development of renal impairment: 7.5% (B) to 3.4% (A); Risk Reduction: 0.45 (CI 0.22–0.94)	Yes. Prescribers reacted 21.1 h sooner to high creatinine levels	Yes, alerts reduced prescribing-related renal impairment by 55%	Yes
Rolfzen<br>2023<br>Secondary Care<br>United States[c]	RCT	1 y<br>July 2020 to June 2021<br>Four hospitals with 11,033 surgical patient discharges in the intervention group and 10,686 in the control	Undetermined/Dose adjustment	To reduce the amount of opioids prescribed to surgical patients at discharge	Alert/Providers who attempted to prescribe higher-than-recommended doses of opioids were automatically notified on the order screen with a suggestion for a reduced prescription. Within the alert, a suggestion to prescribe non-opioid adjuncts was also displayed. Final prescription decisions remained at the discretion of the provider	The ratio of geometric means (active alert/inactive alert) for opioids prescribed at discharge was estimated as 0.95; (95% CI, 0.80 to 1.13; $p = 0.586$); Alerts were not found to affect postdischarge opioid prescriptions with an estimated OR of 1.06 (95% CI, 1.00 to 1.12; $p = 0.052$)	No	NR	Yes
Rolvink[a]<br>2022<br>Secondary Care<br>The Netherlands	Before/After	Duration:12 mo included 73 orders before implementation and 99 orders after implementation<br>Before:<br>February 1, 2018 to July 31, 2018<br>After:<br>August 1, 2018 to January 31, 2019	Interruptive/Drug laboratory alert	Implemented a Clinical Decision Support System (CDSS), recommending dose adjustments upon electronic prescriptions based on renal and hepatic function. The alert provides a dose adjustment proposal and recent laboratory results	Alert/During the intervention period, patient-specific advice was shown to the physician, with the recommended percentage of dose reduction for this patient including the three most recent laboratory results for renal or hepatic function. In both groups, the orders were checked by a pharmacist before preparation of the anticancer drug. When needed a dose adjustment was suggested to the physician. Guidelines for dosage reductions in renal dysfunction are based on the estimated glomerular filtration rate (eGFR) and in our center the CKD-EPI (Chronic Kidney Disease Epidemiology Collaboration) formula is used to calculate the eGFR. Guidelines for dosage reductions in hepatic dysfunction are most frequently based on bilirubin. Therefore, chose bilirubin as a parameter for hepatic dysfunction. The alert was shown to the physician at the moment the order was signed	Included 73 orders before implementation and 99 orders after implementation. Before implementation, 21% of doses were reduced in line with the guidelines vs. 34% after implementation ($p = 0.048$). For hepatic dysfunction, the proportion changed from 11 to 46% $p = 0.011$, while there was no effect for renal dysfunction (24% vs. 26% $p = 0.75$)	Yes	NR	Yes

 © 2025. The Author(s).

Supplementary Appendix 2: (*Continued*)

Author Year Setting Country	Study design/methods	Duration and participants	Type of alert/alert category	Alert objectives	Alert, reminder, or prompt and alert design	Outcomes/results	Beneficial effect on prescribing?	Beneficial effect on patient outcomes?	Can the outcome be attributed to a particular category of alert?
Schwarz[a] 2012 Primary Care United States	RCT	October 2008 and April 2010. Intervention: 17 primary care providers Control: 24 primary care providers	Interruptive/Drug–condition interaction	To evaluate whether computerized clinical decision support (CDS) can increase primary care providers' (PCPs') provision of family planning services when prescribing potentially teratogenic medications	Alert/Forty-one PCPs were randomized to receive one of two types of CDS which alerted them to risks of medication-induced birth defects when ordering potentially teratogenic medications for women who may become pregnant. The 'simple' CDS provided a cautionary alert; the 'multifaceted' CDS provided tailored information and links to a structured order set designed to facilitate safe prescribing. Both CDS systems alerted PCPs about medication risk only once per encounter	Both CDS systems were associated with slight increases in the provision of family planning services when potential teratogens were prescribed, without a significant difference in improvement by CDS complexity ($p = 0.87$). Because CDS was not repeated, 13% of the times that PCPs received CDS they substituted another potential teratogen. PCPs reported significant improvements in several counseling and prescribing practices	No	NR	Yes
Sellier[a] 2009 Secondary Care France	Time series analysis	Six alternating 2-mo control and intervention periods were conducted between August 2006 and August 2007 in two medical departments of a teaching hospital in France. A total of 603 patients and 38 physicians were included	Interruptive/Drug laboratory alert, dose range checking	To determine whether implementing alerts at the time of ordering medication integrated into the computerized physician order entry decreases the proportion of inappropriate prescriptions based on the renal function of inpatients	Alert/During the intervention periods, alerts were triggered if a patient with renal impairment was prescribed one of the 24 targeted drugs that required adjustment according to the estimated glomerular filtration rate (eGFR)	A total of 1,122 alerts were triggered. The rate of inappropriate first prescriptions did not differ significantly between intervention and control periods (19.9% vs. 21.3%; $p = 0.63$). The effect of intervention differed significantly between residents and senior physicians ($p = 0.03$). Residents tended to make fewer errors in intervention vs. control periods (OR: 0.69; 95% CI: 0.41–1.15), whereas senior physicians tended to make more inappropriate prescriptions in intervention periods (OR: 1.88; 95% CI: 0.91–3.89)	No	NR	No

(*Continued*)

 © 2025. The Author(s).

Supplementary Appendix 2: (*Continued*)

Author<br>Year<br>Setting<br>Country	Study design/methods	Duration and participants	Type of alert/alert category	Alert objectives	Alert, reminder, or prompt and alert design	Outcomes/results	Beneficial effect on prescribing?	Beneficial effect on patient outcomes?	Can the outcome be attributed to a particular category of alert?
Sethuraman[a]<br>2015<br>Secondary Care<br>United States	Time series analysis	Duration:<br>5 mo before<br>January 2010 to May 2010<br>5 mo after<br>January 2011 to May 2011<br>7,268 prescriptions before<br>7,292 prescriptions after	Interruptive/Drug allergy interaction; Dose range checking; Drug–drug interaction, Dose adjustment	To test whether the addition of the electronic medication alert system to existing computerized physician order entry reduces the rate and severity of outpatient prescription errors in the pediatric emergency department	Alert/To alert the prescriber to the presence of drug allergies, dose range checking, harmful drug-to-drug interactions, and drug frequency errors in outpatient prescriptions and provide the prescriber with the opportunity to modify the prescription. The prescriber could either modify the prescription or override the alert	There was a significant reduction in the errors per 100 prescriptions (10.4 before vs. 7.3 after; absolute risk reduction = 3.1, 95% CI = 2.2 to 4.0). Drug dosing error rates decreased from 8 to 5.4 per 100 (absolute risk reduction = 2.6, 95% CI = 1.8 to 3.4). There was no decrease in the rate of prescriptions with serious errors (preelectronic medicines administration system [EMAS], 4.9 per 100; post-EMAS, 4.5 per 100; absolute risk reduction = 0.4 per 100, 95% CI = −0.3 to 1.0). Drug–drug interaction errors were not significantly reduced, but drug allergy interaction errors were significantly reduced, declining from 17 to 6 ($p < 0.05$)	Yes	NR	Yes
Shojania<br>1998<br>Secondary Care<br>United States	RCT	9 mo<br>1996/1997<br>396 physicians wrote 5,536 orders for vancomycin for 1,798 patients	Interruptive/None of Page's categories apply	To reduce intravenous vancomycin ordering	Prompt/Screenshots. The intervention showed computerized guidelines for vancomycin ordering at the time of initial vancomycin ordering and after 72 h of therapy. Prescribers are required either to enter an indication or to abort the order. Prescribers were also asked to indicate their reasons for continuing vancomycin use after 72 h of therapy	Initial orders per physician: 9.6 (± 14.5; C); 6.8 (± 9.5; I); $p = 0.03$; 29% less initial prescribing; Renewal orders per physician: 7.0 ± 16.2 (C); 4.5 ± 11.3 (I); 36% less repeat prescribing; Duration of vancomycin therapy: 41.2 d (C); 26.5 d (I); $p = 0.05$; 36% reduction in time	Yes. Reduced vancomycin use in both initial (29% reduction) and repeat prescribing rates (36% reduction). Also prescribed over the shorter period from 41 to 26 d	NR	No
Simon<br>2006<br>Primary Care<br>United States	Time series analysis	2000–2002 (control period)<br>2002–2004 (intervention period)<br>blinded assessment of outcome measure	Interruptive/Formulary alert	Do age-specific alerts and decreased alert burden improve prescribing outcomes?	Alert/See also Smith's paper below. Pop-up alerts displayed in patients >65 only	Making alerts age-specific decreased the alert rate from 18 per physician (B) to 4 (A). This had a Nonsignificant effect on the prescribing of target drugs: Decrease in prescribing rates of target medication per 1,000 prescriptions per quarter: 0.34; $p = 0.75$; (Group sessions linked to introduction of age-specific alerts did not make any difference)	No. Age-specific alerts and subsequent decrease of alert burden did not significantly change prescribing rates	NR	Yes

Supplementary Appendix 2: (*Continued*)

Author<br>Year<br>Setting<br>Country	Study design/methods	Duration and participants	Type of alert/alert category	Alert objectives	Alert, reminder, or prompt and alert design	Outcomes/results	Beneficial effect on prescribing?	Beneficial effect on patient outcomes?	Can the outcome be attributed to a particular category of alert?
Smith<br>2006<br>Primary Care<br>United States	Time series analysis	39 mo:<br>12 mo (C)<br>27 mo (I)	Interruptive/Formulary alert	To reduce prescribing of "potentially contraindicated" agents in elderly	Alert/Alerts of clinically relevant and specific warnings of potentially contraindicated drugs in patients over 65. Alternative drugs are suggested. More information can be accessed if wanted. Interruptive warnings are not applied age specifically and appear in all age groups	Prescribing of target drugs per 1,000 patients:2.2 (B) to 1.7 (A); $p = 0.004$; Trends: Most reduction in amitryptiline/ nortriptyline; no reduction in benzodiazepines	Yes. 22% reduction of nonrecommended drugs in elderly prescribing of benzodiazepines difficult to influence (lack of alternative?)	NR	Yes
Smith[a]<br>2019<br>Primary Care<br>United States	Time series analysis	Duration:<br>1 y between April 2017 and April 2018<br>211,323 office visits for patients with a current opioid prescription and 85,817 office visits for patients with a current benzodiazepine prescription	Interruptive/Drug–drug interaction	To determine the percentage of visits resulting in an opioid prescription for a patient with an active benzodiazepine prescription, and vice versa, before and after the implementation of an electronic health record (EHR) alert notifying clinicians at the time of prescribing of the dangers of concurrent opioid and benzodiazepine use	Alert/Upon initiation of an opioid order, the alert notified clinicians of overlap with an active benzodiazepine prescription, and vice versa; warned of the safety risk, and recommended ordering Naloxone. The clinician could easily dismiss the alert without changing or justifying the prescription order	We observed no significant change in the level of benzodiazepine prescribing immediately following the intervention ($p = 0.56$), and the adjusted trend in benzodiazepine prescribing decreased after the intervention ($p = 0.02$). There was no statistically significant change in the level of opioid prescribing ($p = 0.24$) or in the trend in opioid prescribing after the intervention ($p = 0.80$)	No	NR	Yes
Sonoda[a]<br>2022<br>Secondary Care<br>Japan	Time series analysis	Before implementation M/F (84/49), after implementation M/F (78/52). Median age: before 81, after 79.<br>The PCS was introduced on August 4, 2016. Therefore, two-time segments in the ITS analysis with segmented regression were defined as 52 wk before the PCS implementation (from August 6, 2015, to August 3, 2016) and 52 wk after the PCS implementation (from August 4, 2016, to August 2, 2017)	Passive/Drug laboratory alert	Introduced a prescription checking system (PCS) for in-hospital prescriptions. This retrospective study aimed to evaluate whether a prescription audit by hospital pharmacists using the PCS reduced the rate of dosage errors in renally excreted drugs	Alert/When physicians prescribed the drugs using the computerized physician order entry system, the prescription drug information was sent to the VP-Win total prescription analysis system (TOSHO Inc., Tokyo, Japan). Pharmacists printed the in-hospital prescriptions using the VP-Win system (If the label "renal" had been added in front of the name of the renally excreted drug on the in-hospital prescription, pharmacists also printed the check sheet of dosages according to kidney function using the VP-Win total prescription analysis system. Pharmacists checked the dosages of renally excreted drugs using the PCS at the time of dispensation. If the dosage of the target drugs was appropriate, pharmacists dispensed the drugs. If the dosage of the target drugs was inappropriate, pharmacists asked the prescriber about the prescription contents before dispensing the drugs	Before and after PCS implementation, 474 and 331 prescriptions containing one of the targeted drugs, respectively, were generated. The estimated baseline level of the 4-weekly dosage error rates was 34%. The trend before the PCS implementation was stable with no observable trend. The estimated level change from the last point in the pre-PCS implementation to the first point in the PCS implementation was 220% ($p = 0.001$). There was no change in the trend after the PCS implementation	Yes	NR	Yes

(*Continued*)

 © 2025. The Author(s).

Supplementary Appendix 2: (*Continued*)

Author<br>Year<br>Setting<br>Country	Study design/methods	Duration and participants	Type of alert/alert category	Alert objectives	Alert, reminder, or prompt and alert design	Outcomes/results	Beneficial effect on prescribing?	Beneficial effect on patient outcomes?	Can the outcome be attributed to a particular category of alert?
Srikumar[a]<br>2023<br>Secondary Care<br>United States	Before/After	Evaluated the volume of naloxone prescriptions and fills for the preintervention (March to July) and postintervention months (August to December). 918 filled prescriptions, which were matched to the EHR prescription data for analysis	Interruptive/Dose range checking, Drug–condition interaction, Drug laboratory alert, Drug–drug interaction, Formulary alert	To develop and implement an alert in the electronic health record outlining which patients are at higher risk of opioid overdose and should be co-prescribed naloxone. The aim was to increase co-prescribing of naloxone to qualified patients. Also endeavored to evaluate naloxone prescription volume, fill rates, and statewide dispenses before and after alert implementation	Alert/The best practice advisory (BPA) is activated and a pop-up screen prompts the provider to accept or decline a naloxone prescription. If declining, the provider must free-text or select a reason: patient not present, patient declined, or patient lives in a skilled nursing facility. If "patient not present" is selected, the alert is satisfied for that single encounter. For "patient declined" or "lives in a skilled nursing facility," the alert is suppressed for the next 6 mo. The BPA activates for ambulatory prescriptions in all adult inpatient departments and outpatient clinics. Patients receiving end-of-life comfort care are exempt. Regardless of whether the alert activates, all patients remain eligible to receive a naloxone prescription if desired	Found a 2,144% increase in the number of monthly naloxone prescriptions written after the alert became active ($p < 0.01$). There was no statistically significant change in the percentage of fills. There was a 402.8% increase in unique patients statewide with a naloxone dispense after alert implementation ($p < 0.01$)	Yes	NR	No
Steele<br>2005<br>Primary Care<br>United States	Before/After	9 mo:<br>4 mo before<br>5 mo after<br>7,017 mute alerts before;<br>9,274 pop-up alerts after	Interruptive/Drug laboratory alert	Pop-up alerts inform about drug-lab interactions. Aiming at altered prescribing in hypo- and hyperkalemia, nephrotoxicity, thrombocytopenia, and hepatic inflammation	Alert/Content of pop-up alert for drug-laboratory interaction: type of alert (i.e., "nephrotoxicity"), name of the medication, lab results, and suggestions to delete or change medication. Reaction to alert is optional	Dispensing stopped for any alert: 5.4% (C) to 8.3% (I); $p = 0.17$; Dispensing stopped when abnormal results: 5.6% (B) to 10.9% (A); $p = 0.03$; Lab test ordered when missing lab test: 43% (B) to 62% (A); $p < 0.001$; Lab test ordered when no results available: 5% (B) to 10% (A); $p = 0.03$	Yes. Prescribers responded more appropriately to lab abnormalities, ordered more lab tests, and discontinued potentially harmful medication more often	No, the non-significant difference in ADEs between the control and intervention periods. However, the trial was not powered to detect	Yes

Supplementary Appendix 2: (*Continued*)

Author<br>Year<br>Setting<br>Country	Study design/methods	Duration and participants	Type of alert/alert category	Alert objectives	Alert, reminder, or prompt and alert design	Outcomes/results	Beneficial effect on prescribing?	Beneficial effect on patient outcomes?	Can the outcome be attributed to a particular category of alert?
Strom[a]<br>2010<br>Secondary Care<br>United States	RCT	Duration:<br>6 mo<br>August 9 2006 to February 13, 2007<br>985 clinicians in the intervention<br>986 in the control	Interruptive/Drug–drug interaction	To evaluate the effectiveness of a nearly "hard stop" CPOE prescribing alert intended to reduce concomitant orders for warfarin and trimethoprim-sulfamethoxazole	Alert/An automatic electronic hard-stop alert of a trimethoprim-sulfamethoxazole or warfarin order entered into the CPOE system whenever a prescriber placed an order for trimethoprim-sulfamethoxazole with an already-active warfarin order if warfarin was ordered for a patient already taking trimethoprim-sulfamethoxazole, or when ordering both simultaneously. The hard-stop alert appeared as a pop-up window that notified the clinician that the order could not be processed because of a significant potential drug–drug interaction. The specific text of the stop alert read as follows: 'The prescription of warfarin and TMP/Sulfa together is completely prohibited except in cases of urgent need for the TMP/Sulfa. If you are attempting to prescribe warfarin and the patient is already on TMP/Sulfa, discontinue the TMP/Sulfa, and your order for warfarin will be processed. If you are attempting to prescribe TMP/Sulfa and feel that your patient has an urgent need, then contact the inpatient pharmacy and you will be directed as to how to process the order for TMP/Sulfa.' The alert could be overridden by entering in the order the indication of Pneumocystis carinii pneumonia (PCP) prophylaxis or by bypassing the CPOE and calling the pharmacist directly	The proportion of desired responses (i.e., not reordering the alert-triggering drug within 10 min of firing) was 57.2% (111 of 194 hard stop alerts) in the intervention group and 13.5% (20 of 148) in the control group (aOR: 0.12; 95% CI: 0.045–0.33)	Yes	No, the intervention had unintended adverse consequences that were deemed sufficiently serious to warrant discontinuation of the intervention and early termination of the study. This intervention precipitated clinically important treatment delays in 4 patients who needed immediate drug therapy. Nevertheless, the authors state that a "Review of the electronic records of all 4 individuals involved in these events showed that in no case could we identify specific infectious or thrombotic complications that could have been related to the delays in therapy in the adverse event reporting"	Yes

(*Continued*)

 © 2025. The Author(s).

Supplementary Appendix 2: (*Continued*)

Author<br>Year<br>Setting<br>Country	Study design/methods	Duration and participants	Type of alert/alert category	Alert objectives	Alert, reminder, or prompt and alert design	Outcomes/results	Beneficial effect on prescribing?	Beneficial effect on patient outcomes?	Can the outcome be attributed to a particular category of alert?
Tamblyn[a]<br>2012<br>Primary Care<br>Canada	RCT	Duration:<br>2 y<br>September 2008 to July 2010<br>40 doctors representing<br>2,887 patients in the intervention<br>40 doctors representing 2,741 patients in control	Interruptive/Drug–condition interaction, Formulary alert	To determine whether computerized prescribing decision support with patient-specific risk estimates would increase physician response to psychotropic drug alerts and reduce injury risk in older people	Alert/Physicians randomized to the intervention group received a patient-specific risk of injury alert when a patient was prescribed a psychotropic medication that increased the risk of injury. The personalized alert used a published predictive model to estimate the risk of injury based on the patient's age, sex, injury history, presence of cognitive impairment, gait, and balance problems, and doses of selected psychotropic medication. Graphics, in the form of risk thermometers, were created to show physicians the patient's risk of injury in the next 12 mo related to psychotropic medication as well as nonmodifiable characteristics (eg, age, sex). If the physician attempted to reduce the risk of injury by stopping or decreasing the dose of psychotropic medication, the absolute and relative reduction in risk would be shown as an adjustment in the level of the thermometer and a change in the numeric values. If a new psychotropic drug was started or the dose was increased, the absolute and relative increase in the risk would be shown. If no change in medication was instituted (or the risk was increased by a medication change), physicians had to select a reason for the decision from a standardized pick-list (e.g., prescribed by another physician)	The intervention reduced the risk of injury by 1.7 injuries per 1,000 patients (95% CI 0.2/1,000 to 3.2/1,000; $p = 0.02$). There were significant interactions between baseline risk and intervention status for antipsychotics (interaction term: $p = 0.02$) and anticonvulsants (interaction term: $p = 0.03$), with reductions in doses occurring for patients with greater baseline risk	Yes	Yes, the intervention reduced the risk of injury by 1.7 injuries per 1,000 patients ($p < 0.05$) and was greater for patients with a higher baseline risk of injury ($p < 0.05$)	No
Teich<br>2000<br>Secondary Care<br>United States	Time series analysis	8 wk before implementation (October 19, 1993) and 8 wk after implementation.<br>Number of orders out of the total number of orders:<br>1. 41/350 before 766/805 after<br>2. 5,528/263,549 before 363/64,594 after<br>3. 61/68 before 10/73 after<br>4. 842/3,529 before 1,372/2,923 after	Interruptive/Corollary order alert, Formulary alert/Drug–drug interaction, Duplicate order; Dose range checking, dose adjustment	To improve the quality of prescribing in various areas.<br>1. Change H2 blocker prescribing due to cost implications (oral nizatidine or IV ranitidine as agents of choice).<br>2. Decrease overdosed prescribing<br>3. Change the frequency of ondansetron (tds, not qds)<br>4. Increase heparin prescribing linked to bed rest	Alert/CDDS consisting of prescribing alerts. Intervention screen display when physician orders. Alert content seems clear, well set up, and clinically relevant. Reasons explained preferred options, and alternatives given. Very detailed descriptions on paper. Overriding is possible but must give the reason	1. Prescribing of preferred agents: 15.6% (B) to 81.3% (A) $p < 0.001$<br>2. Overdosed prescribing: 2.1% (B) to 0.6% (A) $p < 0.0013$<br>3. Correct frequency prescribing: 6% (B) to 75% (A)<br>4. Heparin prescribing at bed rest: 24% (B) to 47% (A); $p < 0.001$	Yes. Prescribing improved in various areas—prescribing of preferred agents increased from 15% to 81%—overdosed prescribing decreased from 2.1 to 0.6%—correct frequency prescribing increased from 6 to 75%—heparin prescribing at bed rest increased from 24 to 47%	NR	Yes

Supplementary Appendix 2: (*Continued*)

Author<br>Year<br>Setting<br>Country	Study design/methods	Duration and participants	Type of alert/alert category	Alert objectives	Alert, reminder, or prompt and alert design	Outcomes/results	Beneficial effect on prescribing?	Beneficial effect on patient outcomes?	Can the outcome be attributed to a particular category of alert?
Tisdale[a]<br>2014<br>Secondary Care<br>United States	Before/After	Duration: 20 mo<br>October 2008 to October 2011<br>1,200 patients before and after<br>2,400 patients total<br>Before:<br>October 2008 to October 2009<br>After:<br>March 2011 to October 2011	Interruptive/Drug–condition interaction	To reduce the prescribing of medications known to cause drug-induced torsades de pointes (TdP) and decrease the associated risk of QTc interval prolongation in hospitalized patients with known TdP risk factors	Alert/The alert revealed the patient's QTc prolongation risk score, categorized the patient's risk as moderate or high, and indicated the specific risk factors that were contributing to the score. When an order for a QTc interval-prolonging medication was received by the hospital pharmacy, it was entered into the computer by a pharmacist. If the patient had a calculated QTc interval prolongation risk score in the moderate or high range, the computer alert appeared on the screen to the pharmacist entering the order. When the alert appeared, the pharmacist had the following options: overriding the alert and taking no further action; contacting the prescribing physician, alerting him/her to the fact that the patient was at moderate or high risk, discussing risk mitigation strategies such as correction of serum electrolytes, where necessary, and performing more frequent measurement of QTc intervals, and where appropriate, discussing with the prescriber whether the QTc interval-prolonging drug could be discontinued and replaced with therapy with an alternate agent with less or no potential to cause QTc interval prolongation. The prescriber could also recommend that the alert be overridden, with no further action taken	Implementation of the CDSS resulted in a significant reduction in the adjusted OR of QTc interval prolongation in patients in the CCU (OR, 0.65; 95% CI, 0.56–0.89). Implementation of the CDSS did not result in a significant reduction in the adjusted OR of prescribing any QTc interval-prolonging drug (OR, 0.87; 95% CI, 0.77–1.12. However, implementation of the CDSS led to a significant reduction in the adjusted OR of prescribing noncardiac QTc interval-prolonging drugs (OR, 0.79; 95% CI, 0.63–0.91), primarily fluoroquinolone antibiotics and intravenous haloperidol	Yes	Yes, the CDSS was associated with a significant reduction in QTc	Yes
Vanderman[a]<br>2017<br>Primary and Secondary Care<br>United States	Before/After	1 y<br>Before:<br>September 1, 2012 to February 28, 2013<br>After:<br>September 1, 2013 to February 29, 2014<br>A total of 1,539 patients prealert and 1,490 patients postalert were prescribed 1,952 and 1,897 potentially inappropriate medications (PIMs), respectively	Passive/Formulary alert	To identify a medication as potentially inappropriate in patients aged 65 y and older at the point of CPOE. Medications were designated as potentially inappropriate based on the Beers Criteria	Alert/Alerts were flagged with a small message, "use with caution in pts >1/4 65 y" on the medication order, directly below the medication name. The length of the message was limited by the number of available character fields. To highlight the alert, the text was capitalized, underlined, and in a blue font	There was no significant difference in the rate of new PIMs prealert and postalert overall, 12.6 to 12.0% ($p = 0.13$). However, there was a significant reduction in the rate of the top 10 most common newly prescribed PIMs, 9.0 to 8.3% ($p = 0.016$), and resident providers prescribed fewer PIMs during both time periods	Yes	NR	Yes

(*Continued*)

 © 2025. The Author(s).

Supplementary Appendix 2: (*Continued*)

Author Year Setting Country	Study design/methods	Duration and participants	Type of alert/alert category	Alert objectives	Alert, reminder, or prompt and alert design	Outcomes/results	Beneficial effect on prescribing?	Beneficial effect on patient outcomes?	Can the outcome be attributed to a particular category of alert?
Velez[a] 2014 Secondary Care Spain	Before/After	6 mo. Before: March–April 2011 After: March–May 2012 A total of 54 laboratory tests which corresponded to 46 patients	Undetermined/Drug–drug interaction	To analyze the impact of the CPOE alerts on the reduction of errors due to medication interactions in the prescribing process	Alert/Only severe drug interactions included in the hospital formulary were incorporated into the CPOE software. When a physician prescribed a drug that interacted with Oral anticoagulant therapy (OAT), an alert was displayed, informing the clinician of the risks of the concomitant prescriptions and providing clinical advice about handling the situation	Implementation of alerts reduced the number of medication errors caused by drug interactions by 71.4% ($p = 0.02$). The number of errors diminished from 10.5 per mo to 3 when interaction alerts were introduced	Yes.	NR	Yes
Wally[e] 2023 Secondary Care United States	Time series analysis	October 2017 to December 2020 1,289,697 encounters which represent 369,877 patients	Interruptive/Drug–Drug interaction, Drug–Condition interaction, Corollary Order, Dose Range Checking	To determine the impact of clinical decision support (CDS) tool on the rate of opioid prescribing and opioid dose for patients with chronic musculoskeletal conditions	Alert/One alert notifies the prescriber in real-time of the presence of potential risk factors for abuse, misuse, and diversion of prescription opioids based on eight patient risk factors. It provides an alert when a prescriber is initiating an opioid prescription for a patient with a current benzodiazepine prescription or initiating a benzodiazepine prescription for a patient with a current opioid prescription. Another alert triggers when a prescriber initiates an extended-release opioid for an opioid-naïve patient A third alert suggests completion of a standardized pain agreement for patients who have exceeded 90 d of continuous opioid therapy. A fourth alert suggests a naloxone prescription for patients at high risk of overdose	A small but significant decrease in the percentage of encounters that resulted in an opioid prescription (1.6%, $p < 0.001$), but not on an average dose	Yes	NR	No

 © 2025. The Author(s).

Supplementary Appendix 2: (*Continued*)

Author<br>Year<br>Setting<br>Country	Study design/methods	Duration and participants	Type of alert/alert category	Alert objectives	Alert, reminder, or prompt and alert design	Outcomes/results	Beneficial effect on prescribing?	Beneficial effect on patient outcomes?	Can the outcome be attributed to a particular category of alert?
Wu[e]<br>2024<br>Secondary Care<br>United States	Before/After	January 2017 to December 2023<br>23,653 prescribing encounters during the preimplementation phase,<br>26,664 prescribing encounters during the first phase, and 13,119 prescribing encounters during the second phase	Interruptive/Corollary Order, Drug–Drug Interaction, Drug–Condition Interaction; Formulary Alert	To develop and evaluate a CDS tool to promote naloxone co-prescription for high-risk opioids	Alert/In the first phase, the alert nudged prescribers to consider co-prescribing naloxone whenever an attempt to sign an opioid or benzodiazepine prescription was made. Certain patients were identified as being at high risk of overdose from opioids. Four inclusion criteria were established for opioid prescriptions that also met any of the following: (a) having been prescribed a Morphine Milligram Equivalent (MME) dose of 50 or more per day, (b) having a preexisting active benzodiazepine prescription, (c) having a documented history of opioid use disorder or overdose, or (d) having suboxone on their medication list. Best practice advisory triggers in this higher-risk group defaulted to the "Order" option being selected and a strongly worded recommendation to prescribe naloxone was displayed. In cases where patients trigger the BPA, but do not meet the criteria for inclusion in the high-risk group, a modified alert would display. This "low risk" alert defaulted to selecting "Do Not Order" and a reminder to consider naloxone was shown. In the second phase, the alerts significantly narrowed the target population for the CDS by adding patient-specific clinical context, Alerts showed (1) the patient-specific reason(s) that the BPA was triggered, (2) a reminder that the co-prescription of naloxone in such patients is guideline-driven, (3) "Order" always being preselected, and (4) multiple-choice reasons to decline co-prescribing. These reasons include naloxone not being indicated, the patient reporting that they already have naloxone, a discussion of naloxone with the patient not being possible, the patient declining the prescription, or "Other"	Prior to implementation of the BPA, an average of 13.4% [95% CI, 12.9% to 13.8%] of high-risk opioid prescriptions had naloxone provided. After the implementation of version one of the BPA, this rose to 36.4% [95% CI, 35.2% to 37.5%; $p < 0.001$] corresponding to an OR of 3.69 [95% CI, 3.53–3.85]. The second iteration of the BPA saw a further statistically significant increase to 42.7% [95% CI, 40.6% to 44.8%; $p < 0.001$] with an OR of 4.81 [95% CI, 4.57–5.05] relative to the preimplementation period	Yes	NR	No

(*Continued*)

Supplementary Appendix 2: (*Continued*)

Author<br>Year<br>Setting<br>Country	Study design/methods	Duration and participants	Type of alert/alert category	Alert objectives	Alert, reminder, or prompt and alert design	Outcomes/results	Beneficial effect on prescribing?	Beneficial effect on patient outcomes?	Can the outcome be attributed to a particular category of alert?
Yu[a]<br>2010<br>Primary Care<br>United States	Before/After	2 y<br>Before: all of 2002<br>After: all of 2005<br>51 outpatient practices in the greater Boston area that use a common electronic health record (EHR). These included 40 hospital-based clinics, 4 community health centers, and 7 community-based practices. Both primary care and specialty practices were included. Preintervention ($n = 23{,}056$), Postintervention ($n = 42{,}615$)	Passive and Interruptive/Drug–drug interaction (interruptive and passive), Drug–condition interaction (interruptive), Drug laboratory alert (interruptive and passive)	Added alerts based on all Black-box warnings (BBWs) regarding drug–pregnancy interactions along with most drug–drug interactions, most drug-lab monitoring, and some drug–disease interactions. Placed each alert into one of four clinical severity tiers. Level 1 alerts indicate a fatal or life-threatening interaction. Level 2 alerts indicate an undesirable interaction with the potential for serious injury. Level 3 and level 4 alerts indicate the possibility of an undesirable interaction in which a drug should only be used with caution or may require increased monitoring. All alerts judged sufficiently important were implemented in the longitudinal medical record (LMR)	Alert/The longitudinal medical record (LMR) generated alerts using information from each patient's active medication list, problem list, and laboratory results and applied logic rules to identify potential contraindications. The alerts appeared as an on-screen warning that identified the alerted drug and the issues	There was a slightly higher frequency of nonadherence to BBWs after the intervention (4.8% vs. 5.1%, $p = 0.045$). In multivariate analyses, after adjustment for patient and provider characteristics and site of care, medications prescribed during the preintervention period were less likely to violate BBWs compared to those prescribed during the postintervention period (OR 0.67, 95% CI, 0.47–0.96). However, black-box warning violations did decrease after the intervention for BBWs about drug–drug interactions (6.1% vs. 1.8%, $p < 0.0001$) and drug–pregnancy interactions (5.1% vs. 3.6%, $p = 0.01$)	Yes and No	NR	Yes

[a]Studies that were retrieved in the most recent literature search (not in Schedlbauer et al[8]).
[b]This study was not retrieved by Schedlbauer et al,[8] but was in Page et al.[10]
[c]Obtained from the search conducted for another purpose.
[d]Study that was retrieved by a colleague, not part of the search.
[e]Study suggested by a reviewer that falls outside our search dates.

 © 2025. The Author(s).
